# Supplementary material for: Precision ion separation via self-assembled channels
Source: Nat Commun. 2024 Apr 11;15:3160. doi: 10.1038/s41467-024-47083-0 (PMC11009339; doi:10.1038/s41467-024-47083-0)
Supplement: Supplementary file 1 — Supplementary Information [file 41467_2024_47083_MOESM1_ESM.pdf]

Supplementary Information

Shanshan Hong[1], Maria Di Vincenzo[2], Alberto Tiraferri[3], Erica Bertozzi[3], Radosław Górecki[2], Bambar Davaasuren[4], Xiang Li[1], Suzana P. Nunes[1, 2, 5]

[1]Chemistry Program, Physical Science and Engineering Division (PSE), King Abdullah University of Science and Technology (KAUST), Thuwal, Saudi Arabia

[2]Environmental Science and Engineering Program, Biological and Environmental Science and Engineering Division (BESE), King Abdullah University of Science and Technology (KAUST), Thuwal, Saudi Arabia

[3]Department of Environment, Land and Infrastructure Engineering (DIATI), Politecnico di Torino, Corso Duca degli Abruzzi 24, Turin 10129, Italy.

[4]Core Labs, King Abdullah University of Science and Technology (KAUST), Thuwal, Saudi Arabia.

[5]Chemical Engineering Program, Physical Science and Engineering Division (PSE), King Abdullah University of Science and Technology (KAUST), Thuwal, Saudi Arabia

Corresponding Author: Suzana P. Nunes, suzana.nunes@kaust.edu.sa

32 Figures; 7 Tables; and 42 Pages

Chemicals

Trimesoyl chloride (TMC, 98%, TCI Chemistry) was purified by vacuum distillation at 100 °C for 5 hours prior to utilization. Hexakis(6-deoxy-6-amino)-α-cyclodextrin hexahydrochloride (Am6CD•6HCl, 98%), heptakis(6-deoxy-6-amino)-β-cyclodextrin heptahydrochloride (Am7CD•7HCl, 98%), and octakis(6-deoxy-6-amino)-γ-cyclodextrin octahydrochloride (Am8CD•8HCl, 95%) were purchased form CycloLab Cyclodextrin Research and Development Laboratory Ltd.. β-cyclodextrin (β-CD, 97%), methylsulfonyl chloride (≥99%), sodium azide (NaN_3, ≥99.5%), triphenylphosphine (PPh_3, 99%), magnesium chloride hexahydrate ($MgCl_2$•6H_2O, 99%), calcium chloride dihydrate ($CaCl_2$•2H_2O, 99%), nickel chloride hexahydrate ($NiCl_2$•6H_2O, 98%), barium chloride ($BaCl_2$, 98%), tetramethylammonium chloride ($[N(CH_3)_4]^+Cl^-$, (TMA), >98%) and tetraethylammonium chloride ($[N(CH_2CH_3)_4]^+Cl^-$, (TEA), >98%) were from Sigma-Aldrich. Acetic acid (HAc, 99.9%), hydrochloric acid (HCl, 36.5-38.0 %wt), sodium sulfate (Na_2SO4, 99.3%), sodium chloride (NaCl, 99.5%), and potassium chloride (KCl, 99.5%) were provided by Fisher Chemicals. Lithium hydroxide monohydrate (LiOH•H_2O, 98.1%) and manganese chloride tetrahydrate ($MnCl_2$•4H_2O, 98-101%) were purchased from J. T. Baker Ltd.. Sodium methoxide (30 %wt) and magnesium sulfate heptahydrate ($MgSO_4$•7H_2O, 98+%) were provided by AcroSeal Chemicals and Alfar Aesar, respectively. Cadmium chloride ($CdCl_2$, ≥99%) and cobalt chloride hexahydrate ($CoCl_2$•6H_2O, ≥98%) were purchased from Fluka Chemicals. Hexane and lithium chloride (LiCl) were from VMR. Isoparaffinic fluid (IsoparTM G) was obtained from ExxonMobil. All chemicals were used as received except for TMC. NF90 nanofiltration membranes were provided by DuPont, USA. PAN ultrafiltration membranes were purchased from GMT GmbH, Rheinfelden, Germany. Single crystal silicon wafers (100 polished) were used as substrates for depositing the free standing membranes for Grazing-incidence wide-angle X-ray scattering, Atomic force microscopy and X-ray photoemission spectroscopy measurements.

Methods

Characterization techniques

Nuclear magnetic resonance (NMR). ^{1}H NMR and ^{13}C NMR spectra were obtained on a Bruker-500MHz spectrometer, operating at frequencies of 500 MHz for ^{1}H and 126 MHz for ^{13}C,

respectively. The synthesized compounds were dissolved in D_2O with the addition of deuterated hydrochloric acid (DCl). The chemical shift of D_2O corresponding to the residual H_2O signal is referenced at 4.70 ppm in the data analysis.

Cryogenic transmission electron microscopy (Cryo-TEM). For Cryo-TEM analysis, 3 μL of LiOH-Am7CD solution (prepared as described in the subsequent LiOH-AmCD-TMC membrane preparation section) was vitrified with the use of Vitrobot Mark IV (Thermo Fischer Scientific, MA, USA) on a glow-discharged TEM grid (multiple hole diameter and spacing, 20 nm carbon C-Flat) (Protochips, NC, USA). Samples were analyzed with a Titan Krios G-2 CryoTEM microscope at 300 kV of accelerating voltage.

Fourier-transform infrared spectra (FTIR). FTIR spectra were recorded using a Thermo Scientific Nicolet iS20 spectrometer to investigate the chemical composition of the LiOH-Am7CD-TMC membranes. To obtain an adequate sample quantity, free-standing nanofilms were consecutively extracted from the interface between a 1.23%wt Am7CD/0.03M LiOH solution and a 0.2 %w/v TMC Isopar G solution at 3-minute intervals. The collected material was meticulously washed with hexane and water, followed by thorough drying in a freeze-dryer for one day. ATR-FTIR spectra were acquired within the range of 500 to 3600 cm^{-1}, involving 32 scans, and water background signals were subtracted from the obtained spectra.

Atomic force microscopy (AFM). AFM images were acquired by using the Dimension ICON scanning probe microscope. An RFESPA-75 etched silicon probe was used to gently tap the membrane surface in the ambient air with a scan rate of 1Hz. The AFM images were processed using Gwyddion 2.44 SPM software. The free-standing membranes, prepared as described in a subsequent section, were initially floated in water and then carefully torn to create a crack before being transferred onto silicon wafers. Afterward, all samples were thoroughly air-dried at room temperature.

Transmission electron microscopy (TEM). For TEM analysis of self-standing thin films, the formed LiOH-Am7CD-0.05 TMC self-standing film was transported to water and collected from the water surface using glow discharged PELCO ® TEM 200 mesh copper grid with formvar-carbon film (Ted Pella, Inc., CA, USA). Such collected films were dried and stored in a dry cabinet prior to analysis. The TEM analysis was done with Titan ST HR TEM (FEI Company, OR, USA) at 300kV accelerating voltage.

Scanning electron microscopy (SEM). Field Emission SEM (SEM) (Zeiss Merlin Electron Microscope) was used to characterize the surface morphology and thickness of prepared thin film composite membranes and PAN support. The measurements were performed at an accelerating voltage of 5.0 kV and a working distance of 4 mm. Prior to SEM analysis, the samples were dried at room temperature under vacuum conditions and subsequently sputter-coated with a uniform Iridium layer (3 nm) using a Quorum Q300RT sputter coater.

Surface zeta potential. To assess the surface charge characteristics of the prepared membranes, zeta potential measurements were conducted using a SurPASS^{TM} 3 electrokinetic analyzer (Anton Paar, Austria) equipped with an adjustable gap cell designed for planar samples (20 mm × 10 mm). Two distinct KCl electrolyte solutions were employed, i.e., 1 mM and 10 mM. For the 1 mM solution, tests were conducted across a pH range approximately spanning 2.5–10, while with the 10 mM concentration, the investigated pH range extended from 5.5 to 9. Throughout all measurements, the temperature was consistently maintained at 25 °C, and pH adjustments were automatically performed via the instrument's titration unit using 0.05 M NaOH and 0.05 M HCl solutions.

Inductively coupled plasma - optical emission spectrometry (ICP-OES). The concentrations of ions in the permeate liquid were analyzed using ICP-OES (Agilent Technologies 5110). A calibration curve was established using a series of corresponding standard solutions. To verify the accuracy and consistency of the calibration curve, a second solution with known ionic content was analyzed, with the relative error being kept below 3%. The sample solutions were appropriately diluted with a 1%wt HNO_3 solution to ensure that the ionic concentrations fell within the range of 0.2 ppm to 200 ppm.

X-ray photoelectron spectroscopy (XPS). After a two-day vacuum drying period, the free-standing films transferred onto silicon wafers were subjected to further elemental analysis. XPS was carried out utilizing an Axis-Ultra DLD spectrometer with Al Kα radiation (hν=1486.6 eV) under a base pressure of 3×10^{-9} mbar. The binding energy data were calibrated using the C1s signal of aromatic carbon at 284.5 eV as a reference. The C1s spectrum was methodically deconvoluted into several characteristic peaks for detailed analysis.

Powder X-ray diffraction (PXRD). The 2D PXRD measurement of crystalline Am7CD powder (preparation details provided in the subsequent section titled 'Single crystal growth and preparation of bulk polycrystalline powder') was conducted using ground crystals. This analysis was

performed on a Bruker D8 Discover system equipped with IuS microfocus Cu-radiation (λ = 1.54184 Å) and an Eiger2R_500K detector. The 1D XRD pattern was generated by integrating the data obtained from the 2D frame

X-ray crystallography. A suitable sized single crystal was probed on a Bruker D8 Venture diffractometer equipped with an IuS microsource and a Photon II detector at a temperature of 120 K. Molybdenum (Mo) was used as the X-ray source with Kα radiation (λ = 0.71073Å). The crystal structure was initially solved with the SHELXT structure solution program using Dual Space and subsequently refined with the SHELXL implemented in Olex2 program package. All non-hydrogen atomic positions were refined anisotropically. The H-atoms were treated as riding with Uiso(H) values set at 1.2Ueq(C) for tertiary CH and secondary CH_2-groups and 1.5Ueq(C) for OH. Notably, the structure contains two voids housing heavily disordered H_2O-solvent molecules. Consequently, a solvent mask was applied during the final stages of the least-squares refinement. Supplementary Table 1 and 2 provide detailed structural information for $C_{42}H_{77}N_7O_{28}$·[solvents]. The crystal structure was validated using the checkCIF and no A- or B-level alerts were generated.

Supplementary Table 1. Hydrogen Bonds for $C_{42}H_{77}N_7O_{28}$·[solvents].

D	H	A	d(D-H)/Å	d(H-A)/Å	d(D-A)/Å	D-H-A/°
**Intramolecular**						
O1	H1	O2	0.84	1.88	2.704(1)	165.2
O3	H3	O4	0.84	1.92	2.743(1)	167.2
O5	H5	O6	0.84	1.91	2.739(1)	167.1
O7	H7	O8	0.84	2.03	2.831(1)	160.0
O9	H9	O10	0.84	1.87	2.656(1)	156.0
O11	H11	O12	0.84	1.90	2.725(1)	165.7
O13	H13	O14	0.84	1.96	2.691(1)	144.9
**Intermolecular**						
O2	H2	N2[1]	0.84	1.83	2.658(1)	167.4
O4	H4	N3[1]	0.84	1.84	2.675(1)	177.8
O6	H6	N4[1]	0.84	1.89	2.696(1)	159.2
O8	H8	N5[1]	0.84	1.85	2.684(1)	176.4
O10	H10	N6A[1]	0.84	1.85	2.670(4)	165.8
O12	H12	N7[1]	0.84	1.84	2.667(1)	168.8
O14	H14	N1A[1]	0.84	1.84	2.656(3)	164.2
C8	H8A	O10[2]	1.00	2.58	3.268(1)	125.7
C9	H9A	O9[2]	1.00	2.61	3.606(1)	177.3
C9	H9A	O10[2]	1.00	2.65	3.237(1)	117.7

C18	H18	O14[3]	1.00	2.57	3.234(1)	123.3
C19	H19	O14[3]	1.00	2.59	3.199(1)	119.3
C39	H39A	O23	0.99	2.66	3.533(1)	147.5
C36	H36B	O7[4]	0.99	2.49	3.358(9)	146.4
C41A	H41A	O25	0.99	2.63	3.490(2)	144.7
C36A	H36C	O27	0.99	2.60	3.474(17)	147.4
C41	H41D	O3[5]	0.99	2.41	3.326(5)	152.8

[1] -1+*x*, +*y*, +*z*; [2] +*x*, -1+*y*, +*z*; [3] +*x*, +*y*, -1+z; [4] 1+*x*, +*y*, 1+*z*; [5] 1+*x*, 1+*y*, +*z*

Supplementary Table 2. Crystal data and structure refinement for $C_{42}H_{77}N_7O_{28}$·[solvents].

Empirical formula	$C_{42}H_{77}N_7O_{28}$
Formula weight	1125.28
Temperature/K	120.00
Crystal system	triclinic
Space group	*P*1
a/Å	8.4022(5)
b/Å	14.9737(9)
c/Å	15.4144(9)
α/°	101.239(3)
β/°	98.236(3)
γ/°	99.509(2)
Volume/Å^3	1844.88(19)
Z	1
ρ_{calc} g/cm^3	1.013
μ/mm^{-1}	0.085
F(000)	599.0
Crystal size/mm^3	0.599 × 0.524 × 0.442
Radiation	MoKα (λ = 0.71073)
2Θ range for data collection/°	2.83 to 64.172
Index ranges	-12 ≤ h ≤ 12, -22 ≤ k ≤ 22, -23 ≤ l ≤ 23
Reflections collected	104060
Independent reflections	25289 [R_{int} = 0.0496, R_{sigma} = 0.0414]
Data/restraints/parameters	25289/19/717
Goodness-of-fit on F^2	1.044
Final R indexes [I>=2σ (I)]	*R1* = 0.0290, *wR2* = 0.0801
Final R indexes [all data]	*R1* = 0.0301, *wR2* = 0.0809
Largest diff. peak/hole / e Å^{-3}	0.20/-0.19
Flack parameter	0.1(2)

CCDC deposition number: 2290245

Preparation of LiOH-AmCD-TMC thin film composite (TFC) membranes

86.7 mg of either commercial hexakis(6-deoxy-6-amino)-α-cyclodextrin hexahydrochloride (Am6CD•6HCl), heptakis(6-deoxy-6-amino)-β-cyclodextrin heptahydrochloride (Am7CD•7HCl) or octakis(6-deoxy-6-amino)-γ-cyclodextrin octahydrochloride (Am8CD•8HCl) was individually dissolved in 7mL of 0.030M LiOH solution. The resulting suspensions were then ultrasonicated for 20 seconds and left to incubate overnight (Namely LiOH-AmCD solution, 1.23%wt). The pH was approximately 8.4, as detected by a pH meter (mod. Accumet AB150, Fisher™). Subsequently, a commercial polyacrylonitrile (PAN) support layer was fixed in a polytetrafluoroethylene frame and immersed in the per-amino CD aqueous solution for 10 minutes. After removing the solution and eliminating the excess water with a rubber roller, a solution of TMC in Isopar G with a specific concentration was applied to the support surface and allowed to react for 3 minutes. The membrane was then washed five times with hexane to remove residual TMC solution and subjected to a 10-minute thermal treatment at 80 °C. The membrane was air-dried overnight at room temperature and stored in Mili-Q water at 4 °C before testing. The resulting membranes, varying in per-amino CD type and TMC concentrations, are detailed in Supplementary Table 3.

Supplementary Table 3. Fabrication conditions of LiOH-AmCD-TMC membranes

Membrane code	cyclodextrin type	TMC concentration (%w/v)	Reaction time (minute)
LiOH-Am6CD-0.1 TMC	Am6CD	0.1	3
LiOH-Am8CD-0.1 TMC	Am8CD	0.1	3
LiOH-Am7CD-0.0025 TMC	Am7CD	0.0025	3
LiOH-Am7CD-0.005 TMC	Am7CD	0.005	3
LiOH-Am7CD-0.01 TMC	Am7CD	0.01	3
LiOH-Am7CD-0.05 TMC	Am7CD	0.05	3
LiOH-Am7CD-0.075 TMC	Am7CD	0.075	3
LiOH-Am7CD-0.1 TMC	Am7CD	0.1	3
LiOH-Am7CD-0.2 TMC	Am7CD	0.2	3
LiOH-Am7CD-0.3 TMC	Am7CD	0.3	3

Synthesis of heptakis(6-deoxy-6-amino)-β-cyclodextrin (Am7CD)

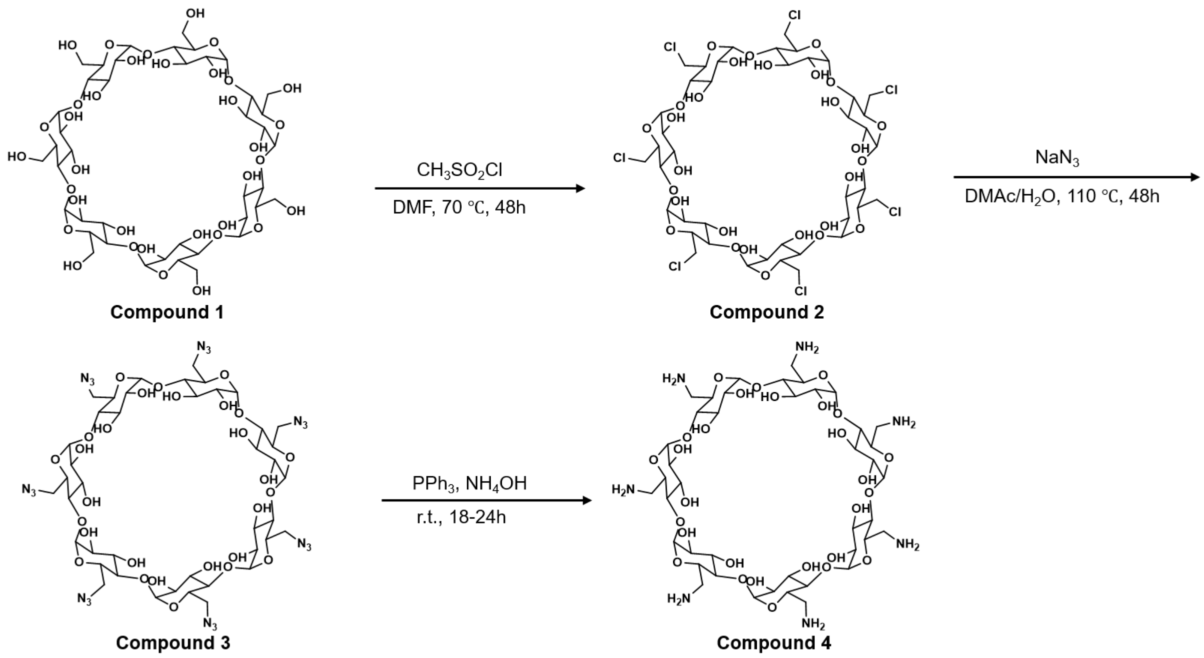

Supplementary Figure 1. Synthesis of Am7CD.

The synthesis procedure follows Supplementary Fig. 1 and was adapted from the existing literature[1-4].

Step 1: Synthesis of heptakis(6-deoxy-6-chloro)-β-cyclodextrin (Cl-βCD)

The synthesis of Cl-βCD began with the addition of 8.3g β-CD to 150 mL ultra-dry DMF under a nitrogen atmosphere. Methylsulfonyl chloride (23.3 mL) was then introduced dropwise into the cyclodextrin solution with continuous stirring. The reaction proceeded for 48 hours at 70 °C, followed by solvent rotary evaporation at 80 °C. The residual material was dissolved in methanol and stirred for 30 minutes. Subsequently, a 30% wt sodium methoxide solution in methanol was added dropwise until a pH of about 7 was reached. After an additional 4 hours of stirring, the resulting suspension was poured into ice water. The precipitate obtained after centrifugation was washed with methanol until the supernatant became colorless. The precipitate was finally dried in an oven at 50 °C, yielding 7.3 g of Cl-βCD.

Step 2: Synthesis of heptakis(6-deoxy-6-azido)-β-cyclodextrin (N_3-βCD)

4.23 g of Cl-βCD was dissolved in 200 mL DMAc and 26.7 mL water, followed by the addition of 4.77 g NaN_3 under an N_2 balloon. Then the temperature increased to 110 °C for 2 days. The

resulting light orange solution underwent further concentration using a rotary evaporator at 80 °C. Upon cooling to room temperature, the concentrated solution was carefully poured into a substantial volume of water at 0 °C, yielding a precipitate that was subsequently filtered. Then, water was used to thoroughly wash out any unreacted NaN_3, followed by a single rinse with acetonitrile. The resulting material was then dried at 50 °C under high vacuum conditions, yielding N_3-βCD in the form of a white to yellow powder (4.2 g).

Step 3: Synthesis of heptakis(6-deoxy-6-amino)-β-cyclodextrin (Am7CD)

14.1 g PPh_3 was added to a solution of 3.02 g N_3-βCD in 56 mL anhydrous DMF, followed by stirring for 3.5 hours until the bubbling ceased. The solution became turbid slowly when 21 mL concentrated ammonia (30%wt) was added dropwise. The suspension was maintained at room temperature for over 20 hours and then concentrated via a rotary evaporator at 50 °C. The concentrate was poured into a substantial amount of ethanol to precipitate. The precipitate was separated by filtration and washed thoroughly with ethanol and acetone to remove any unreacted PPh_3. The isolated solid was dried at 50 °C under high vacuum for 24 hours to obtain a light-yellow powder (1.65 g).

^{1}H NMR (500 MHz, D_2O) δ 5.12 (d, J = 3.5 Hz, 1H), 4.16 (ddd, J = 10.4, 7.3, 3.3 Hz, 1H), 3.93 (d, J = 9.5 Hz, 1H), 3.66 – 3.60 (m, 1H), 3.54 (t, J = 9.4 Hz, 1H), 3.40 (dd, J = 13.6, 3.4 Hz, 1H), 3.26 – 3.18 (m, 1H).

^{13}C NMR (126 MHz, D_2O) δ 101.37, 82.10, 72.09, 71.56, 67.71, 40.12.

Preparation of HAc-Am7CD-TMC thin film composite (TFC) membranes

144.0 mg synthesized Am7CD was added into 14mL of distilled water. 16 µL acetic acid was then added to the suspension, resulting in a clear solution with a pH of approximately 7.4 during sonication (referred to as HAc-Am7CD solution, 9.1 mmol/L). A PAN support was immersed in this aqueous solution for 10 minutes, and any excess solution on the surface was removed using a rubber roller. Then, an organic solution containing TMC (0.3%w/v) was poured on the soaked support and allowed to react for 12 minutes. After washing with hexane, the membrane was dried in an oven at 80 °C for 10 minutes. The membrane was air-dried overnight at room temperature to remove any residual Isopar G. The resulting membranes were subsequently soaked in distilled water until testing. It is worth highlighting that all HAc-Am7CD-0.3 TMC TFC membranes utilized in filtration measurements, surface z-potential and SEM characterization, as well as the

free-standing membrane samples used for XPS, GIWAXS and AFM analyses, were manufactured with a 12-minute reaction time. The extended reaction time of 28 minutes was exclusively applied to the free-standing membrane samples intended for TEM measurements. This prolonged reaction time was implemented to increase membrane thickness and prevent damage caused by the electron stream during TEM analysis.

Preparation of free-standing membrane samples

The aqueous solution containing amino cyclodextrin (at the same concentration as in the previous sections on TFC membrane preparation) was poured into a glass vial, followed by gently introducing the organic solution containing TMC along the inner wall of the vial. After a specified reaction time (3 minutes for LiOH-Am7CD-TMC membranes; 12 minutes or 28 minutes for HAc-Am7CD-TMC membranes), the solutions were extracted using a syringe, and the resulting film at the liquid-liquid interface was deposited onto a pre-placed silicon wafer substrate in a vial. Subsequently, hexane was employed for rinsing the membrane at least five times to remove residual acyl chloride. The membrane was then floated on water to eliminate unreacted amino cyclodextrin before being transferred to various substrates, including silicon wafers for Grazing-Incidence Wide-Angle X-ray Scattering, Atomic Force Microscopy, and X-ray Photoelectron Spectroscopy measurements, as well as PELCO ® TEM 200 mesh copper grids with Formvar-carbon film for Transmission Electron Microscopy analysis.

Single crystal growth and preparation of bulk Am7CD polycrystalline powder

Two identical solutions were prepared individually by dissolving 86.7 mg of commercial Am7CD•7HCl in 7 mL of 0.03 M LiOH solution, followed by 20 seconds of ultrasonication. Then, the resulting yellowish solution was filtered using polyamide filters with a pore size of 0.45 μm. Subsequently, one of the solutions was sealed and left undisturbed on a flat surface at room temperature (20 °C) for 1-2 days to allow the formation of micrometer-scale crystals. The other solution was stored overnight, ultrasonicated for 1 hour, and left undisturbed for 8 hours until the clear solution became milky. The suspension was finally centrifuged, washed five times with water, rinsed with ethanol, and dried in an oven at 80 °C.

Filtration tests of neutral solutes

Various neutral organic compounds with different Stokes radii (R_S) were used to study the pore size of the different types of cyclodextrin-based composite membranes. The selected solutes were ethylene glycol (62.07 Da), glycerol (92.09 Da), xylose (150.13 Da), glucose (180.16 Da), sucrose (342.30 Da), and raffinose (504.42 Da). The Stokes radius was calculated based on Equation 1[5]. The calculated Stokes radius for raffinose, sucrose, glucose, xylose, glycerol, and ethylene glycol are 0.539 nm, 0.462 nm, 0.359 nm, 0.334 nm, 0.275 nm, and 0.235 nm, respectively. Filtration tests were performed using single-solute feed solutions and a concentration of 100 ppm under 5 bar of applied pressure. After each filtration cycle at room temperature (20°C), the first 15 mL of permeates were discarded and the samples were collected for the analysis of total organic carbon content, whereas the rejection rate was calculated using Equation 2. Here, C_P represents the total organic carbon content in the permeate, while C_F denotes the total organic carbon content of the feed. Please note that unless otherwise clarified, the error bars in all figures and supplementary figures represent the standard deviation of a sample, derived from a minimum of three independent experiments.

$$\log_{10}(R_s) = -1.3363 + 0.395 \times \log_{10}(M_w) \quad (1)$$

where R_s is in nm and molecular mass M_w is in $g \cdot mol^{-1}$.

Ion sieving nanofiltration in a dead-end filtration system

Ion sieving nanofiltration tests were performed using Sterlitech stainless steel cells (model HP4750) with a membrane effective area (S) of 13.8 cm². The tests were conducted at a pressure of 5 bar and room temperature (20°C), unless otherwise specified. The membrane were evaluated using two types of feeds: (i) single-salt solutions, each containing a specific salt ($MgCl_2$, LiCl, $CaCl_2$, NaCl, KCl, Na_2SO_4, $MgSO_4$, $NiCl_2$, $MnCl_2$ or $BaCl_2$, $CdCl_2$, $CoCl_2$, $[N(CH_3)_4]^+Cl^-$ and $[N(CH_2CH_3)_4]^+Cl^-$) at a consistent concentration of 2000 ppm (the only exception was HCl, presented as a diluted solution with a pH of 3.8); (ii) binary-salt solutions with varying $MgCl_2$/LiCl mass ratios, specifically: $MgCl_2$/LiCl = 2000ppm/100ppm, 2000ppm/50ppm, 2000ppm/33ppm, 2000ppm/25ppm, 4000ppm/200ppm or 6000ppm/300ppm.

The tested membrane was initially compacted at 6 bar for 1 to 2 hours until the steady-state was reached. Subsequently, the applied pressure was reduced to 5 bar to perform filtration tests using

saline solutions. After discarding the initial 10 mL of permeate, the samples were collected. The salt rejection rate (R) was calculated using Equation 2, where C_P and C_F represent the ion concentrations in the permeate and feed solutions, respectively. In single-salt filtration tests, ion concentrations were determined by conductivity measurements using a conductivity meter (Eutech, mod. CON 2700). This involved establishing a relationship between ion concentration and electrical conductivity using known standards. As a result, we could determine ion concentrations in the sample solutions based on their measured conductivities. For filtration tests involving binary-salt feed compositions, ion concentrations in the liquid samples were directly measured using ICP-OES (Agilent Technologies, 5110). To assess the membrane selectivity, the separation factor (SF) was calculated using Equation 3, where $C_{Mg,\,F}$, $C_{Li,\,F}$, are the concentration of Mg^{2+} and Li^+ in the feed, respectively; while $C_{Mg,\,P}$, $C_{Li,\,P}$ represent the concentration of Mg^{2+} and Li^+ in the permeate. A higher SF value typically indicates greater selectivity. Flux (L m^{-2} h^{-1}) and permeance (L m^{-2} h^{-1} bar^{-1}) were calculated using Equation 4 and Equation 5, respectively. Here, V (L) was monitored via a computer-interfaced balance and it represents the total volume of the permeating fluid during a specific time interval (Δt, h) at the applied pressure (ΔP, bar).

$$R\,(\%) = (1 - \frac{C_P}{C_F}) \times 100\% \tag{2}$$

$$SF = \frac{C_{Mg,\,F}/C_{Li,\,F}}{C_{Mg,\,P}/C_{Li,\,P}} \tag{3}$$

$$J = \frac{V}{S \times \Delta t} \tag{4}$$

$$A = \frac{J}{\Delta P} \tag{5}$$

Low-pressure cross-flow filtration of diluted brines

Pure water permeance, permeate flux, and solute rejection were evaluated in a Sterlitech cross-flow laboratory-scale filtration system (WA, USA) comprising three stainless steel cells in series, as illustrated in Figure 4b of the main text. The housing cells consist of a rectangular channel with an active membrane area of 20.6 cm^2. In total, this gives 61.8 cm^2 of membrane area being tested, which provides a more reliable statistical analysis. The cross-flow filtration system comprises a high-pressure feed flow pump (Hydracell, Wanner Engineering, Inc., Minneapolis, MN), a

stainless steel feed vessel, three flat membrane housing cells (model CF016), temperature control and data acquisition systems. The cross-flow rate in the filtration system was continuously monitored using a floating disc rotameter. Adjustments to this rate, as well as to the operating pressure, were achieved through the coordinated use of a bypass valve and a back-pressure regulator (Swagelok, Solon, OH). Additionally, the permeate flow rate was automatically recorded at 60-second intervals using a computer-interfaced balance, ensuring precise and consistent data collection. The temperature was controlled via a recirculating chiller (DuraChill, Polyscience, USA) with a stainless-steel coil immersed in the feed tank.

Prior to each experiment, the membrane was immersed in water overnight. Following the loading of the membrane sample into the cross-flow housing cell, the sample was compacted for 4 h at an applied pressure of 20 bar. The applied pressure was then lowered to a value of 5 bar, 10 bar, or 18 bar, respectively. The pure water flux, $J_{w,0}$, was calculated by dividing the volumetric permeate rate obtained at steady-state by the membrane area based on Equation 4, and the pure water permeability coefficient of the membrane, "A", was calculated from the value of $J_{w,0}$ (Equation 5). Solute rejection tests were performed with a constant cross-flow velocity of 0.50 m/s, and the feed stream consisted of a diluted salt lake brine (the composition is presented in Supplementary Table 4). The total volume of the feed solution for each rejection test was 5 L and the overall duration of each rejection experiment was roughly 8 h. Each collected feed and permeate sample was analyzed by means of ICP-OES to determine the single ion observed rejections using Equation 2 and separation factor using Equation 3.

High-pressure cross-flow filtration of concentrated brine

To assess the performance of Am7CD membranes in practical Li^+/Mg^{2+} separation, three synthetic feed solutions closely mimicking the composition of real lithium-containing brines were adopted. These solutions included concentrated seawater (typical brine from RO seawater desalination activities), diluted Imperial geothermal brine (diluted by a factor of 7), and diluted Lungmu Co salt-lake brine (diluted by a factor of 4), which are characterized by various Li^+ levels and composition. Each synthetic feed solution used in the measurements was prepared by dissolving the proper amount of analytical grade reagents in deionized water (D.I. water, Milli-Q® IX 7005, Merck Millipore, Darmstadt, Germany), as detailed in Supplementary Table 5. All separation experiments were performed with a cross-flow lab-scale system described in detail in our previous

publications[6,7]. It comprises a high-pressure pump (Hydra-cell pump, Wanner Engineering, Inc., Minneapolis, MN), feed reservoir, membrane housing cell, temperature control, and data acquisition system. The housing cell consists of a plate-and-frame unit with a 7.7 cm long, 2.7 cm wide, and 0.3 cm high rectangular channel, resulting in a 21 cm^2 total active area of the membrane sample. In this experimental setup, the retentate stream was continuously recirculated back to the feed reservoir. Meanwhile, the permeate stream was collected in a vessel placed on a computer-interfaced balance, allowing for the calculation of water flux across the membrane by measuring the change in permeate volume over time. The crossflow rate was closely monitored using a flowmeter (model 1900, ASA, Sesto San Giovanni, Italy) and adjusted, along with the operating pressure, through a bypass valve and a back-pressure regulator (Swagelok, Solon, OH). Temperature levels were constantly monitored using a probe thermometer and maintained through a recirculating chiller (Model MC 1200, Lauda, Lauda-Königshofen) equipped with a stainless-steel coil submerged in the feed tank.

Separation experiments were conducted with adjustments to the operating conditions tailored to each synthetic feed solution. Throughout all tests, the feed temperature was maintained at 25°C, and the cross-flow rate was set at 5 L/h. The applied pressure, however, varied depending on the specific synthetic solution, specifically 70 bar for concentrated seawater and 60 bar for Imperial geothermal brine and Lungmu Co salt-lake brine. At these operating pressures, each membrane underwent initial compaction using D.I. water until the permeate flux reached a stabilized state, typically overnight. Once the flux reached a steady state, the pure water flux (J_w) was determined using Equation 4.

Following the compaction stage, the synthetic feed solutions were introduced into the feed tank, and an initial feed sample was collected. The overall rejection performance of Am7CD membranes was evaluated by periodically collecting permeate and feed (recirculated retentate stream) samples as the recovery rates varied. Moreover, every collected feed and permeate sample underwent analysis via ICP-OES to determine the rejections of individual ions according to Equation 2, and separation factors were calculated using Equation 3. Notably, the endpoint of the separation experiments differed for concentrated seawater compared to the other two feed solutions. Specifically, for the former, the test was terminated once oversaturation and salt precipitation commenced (typically occurring at recovery rates higher than 20-25%). Conversely, for the other

brines, the endpoint was determined based on achieving either 50% recovery or fluxes dropping below 3 L m^{-2} h^{-1}, depending on which condition was met first.

Supplementary Table 4. Composition of the synthetic feed solutions used in low-pressure cross-flow filtration tests.

Ion	Concentration (g/L)	Salt	Concentration (g/L)
Li^+	0.030	LiCl	0.182
Na^+	0.3	NaCl	0.763
K^+	0.3	KCl	0.573
Mg^{2+}	1.50	$MgCl_2$	5.94
Ca^{2+}	0.030	$CaCl_2$	0.083

Supplementary Table 5. Composition of the synthetic feed solutions, i.e., concentrated seawater, Imperial geothermal brine, and Lungmu Co salt-lake brine: ion concentrations and their ratios in relation to lithium.

	Concentrated seawater		Geothermal brine Imperial		Salt-lake brine Lungmu Co	
	Concentration (g/L)	$M^{n+/-}$ /Li^+	Concentration (g/L)	$M^{n+/-}$ /Li^+	Concentration (g/L)	$M^{n+/-}$ /Li^+
Li^+	0.140	1.00	0.047	1.00	0.042	1.00
Na^+	20.8	149	9.36	200	9.20	219
K^+	0.690	4.93	1.78	38.1	n.d.	n.d.
Mg^{2+}	2.25	16.1	0.057	1.22	3.60	85.7
Ca^{2+}	0.390	2.79	3.39	72.5	0.280	6.67
Ba^{2+}	n.d.	n.d.	0.323	6.91	n.d.	n.d.
Cl^-	37.2	266	22.6	485	25.4	102
SO_4^{2-}	4.73	33.8	n.d.	n.d.	n.d.	n.d.
Total dissolved solids (g/L)	66.2		37.6		38.5	

n.d.: not determined

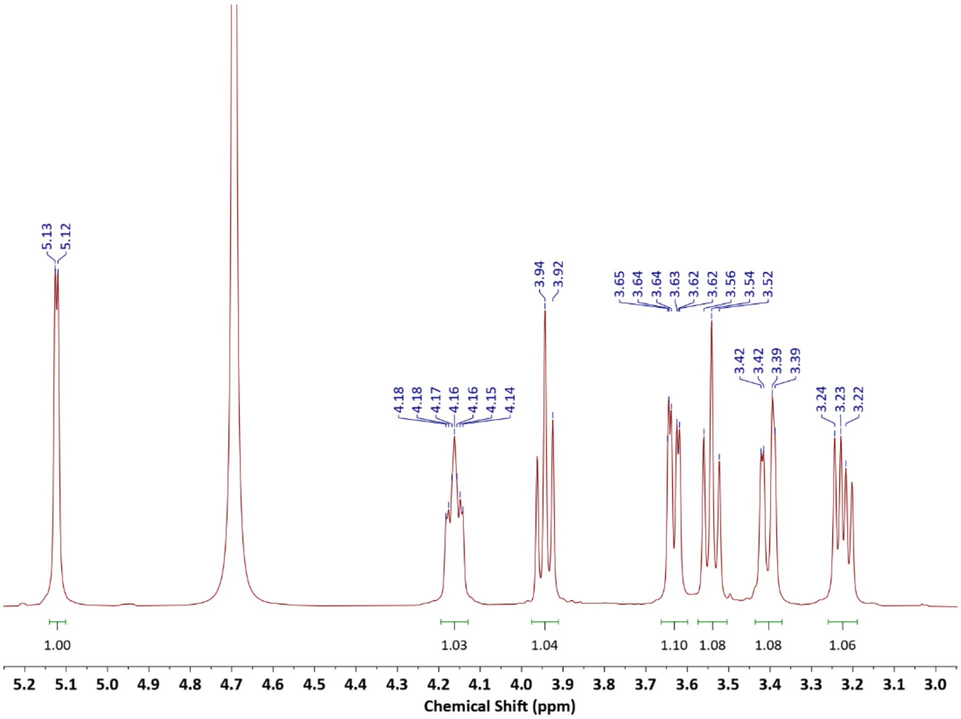

Supplementary Figure 2. ^{1}H NMR spectrum of synthesized Am7CD in D_2O with added deuterated hydrochloric acid (DCl).

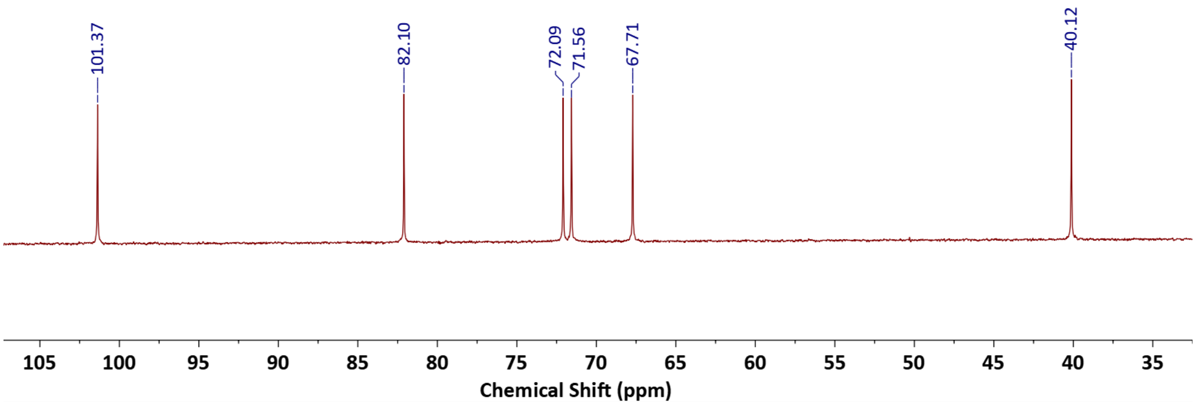

Supplementary Figure 3. ^{13}C NMR spectrum of synthesized Am7CD in D_2O with added DCl.

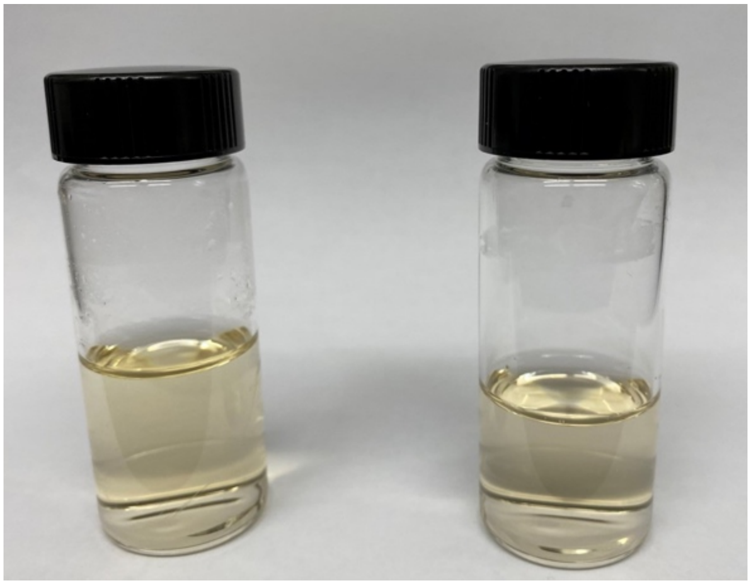

Supplementary Figure 4. Comparison between LiOH-Am7CD solution (86.7 mg Am7CD•7HCl dissolved in 7 mL 0.030M LiOH solution, 9.0 mmol/L, left) and HAc-Am7CD solution (144.0 mg synthesized Am7CD dissolved in 14 mL water with 16 µL acetic acid, 9.1 mmol/L, right).

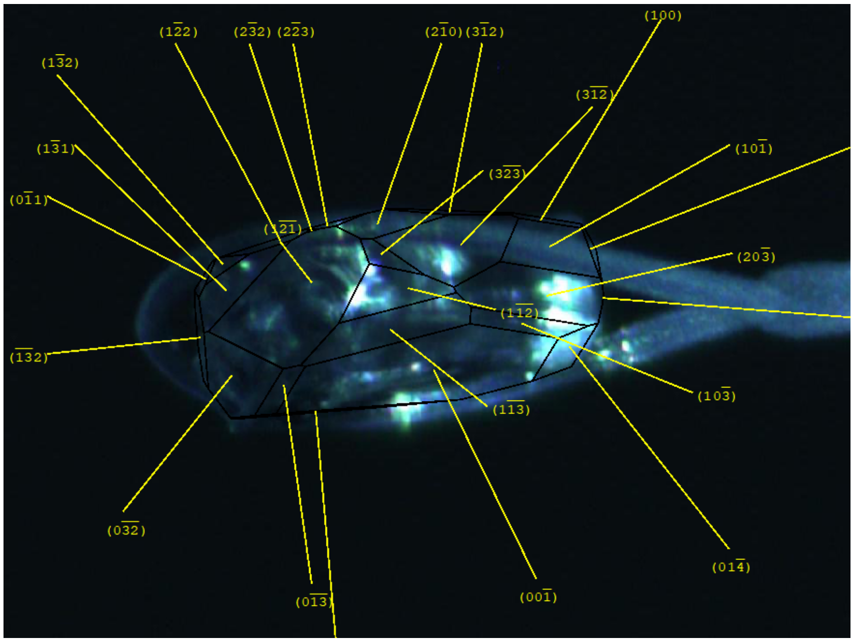

Supplementary Figure 5. Digital photo of the isolated LiOH-Am7CD single crystal.

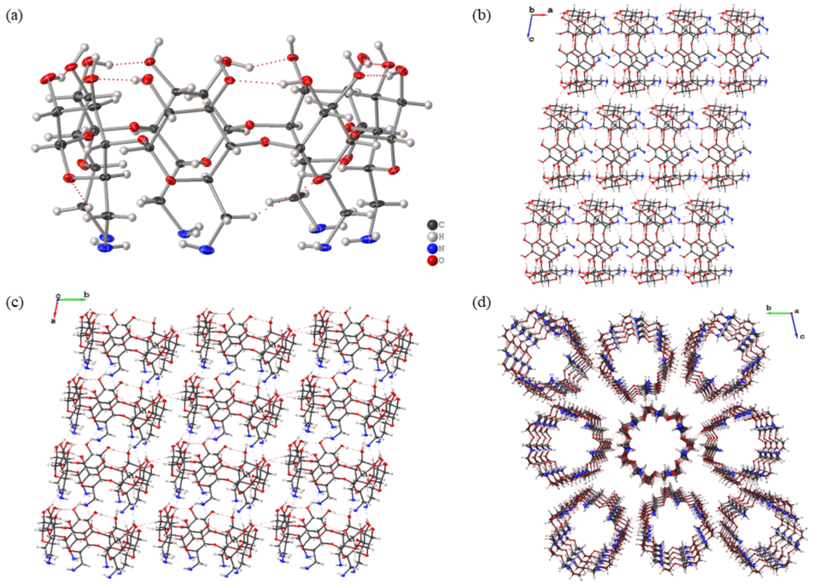

Supplementary Figure 6. (a) The LiOH-Am7CD molecule in the asymmetric unit and (b-d) packing arrangements of the molecules viewed along all the crystallographic axes. The thermal ellipsoids are drawn at 50 % probability level. The intra- and intermolecular H-bonds are illustrated by dotted-red lines.

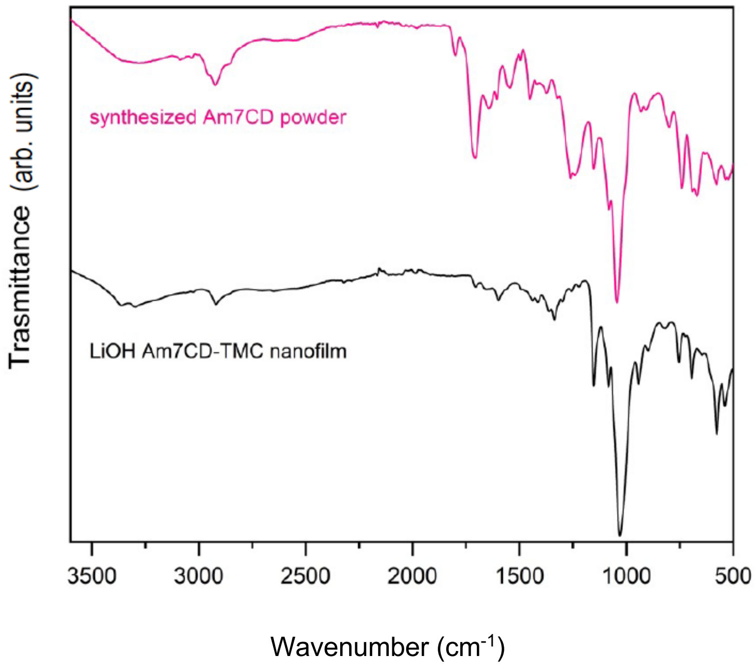

Supplementary Figure 7. Comparison of FTIR spectra of Am7CD monomer powder and isolated free-standing LiOH-Am7CD-TMC nanofilm obtained reacting 1.23% wt LiOH-Am7CD aqueous solution and 0.2% w/v TMC solution in Isopar G at 3 minutes intervals.

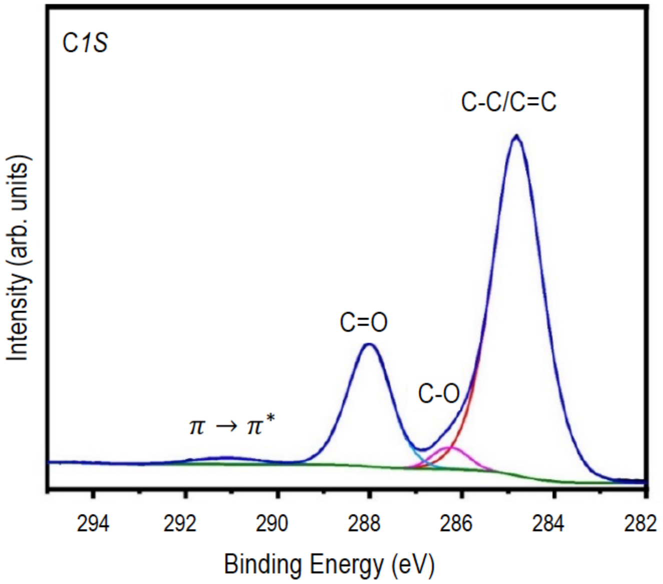

Supplementary Figure 8. Deconvolution of the C1*s* spectrum in high resolution X-ray photoelectron spectroscopy for the LiOH-Am7CD-0.01 TMC membrane deposited on a silicon wafer.

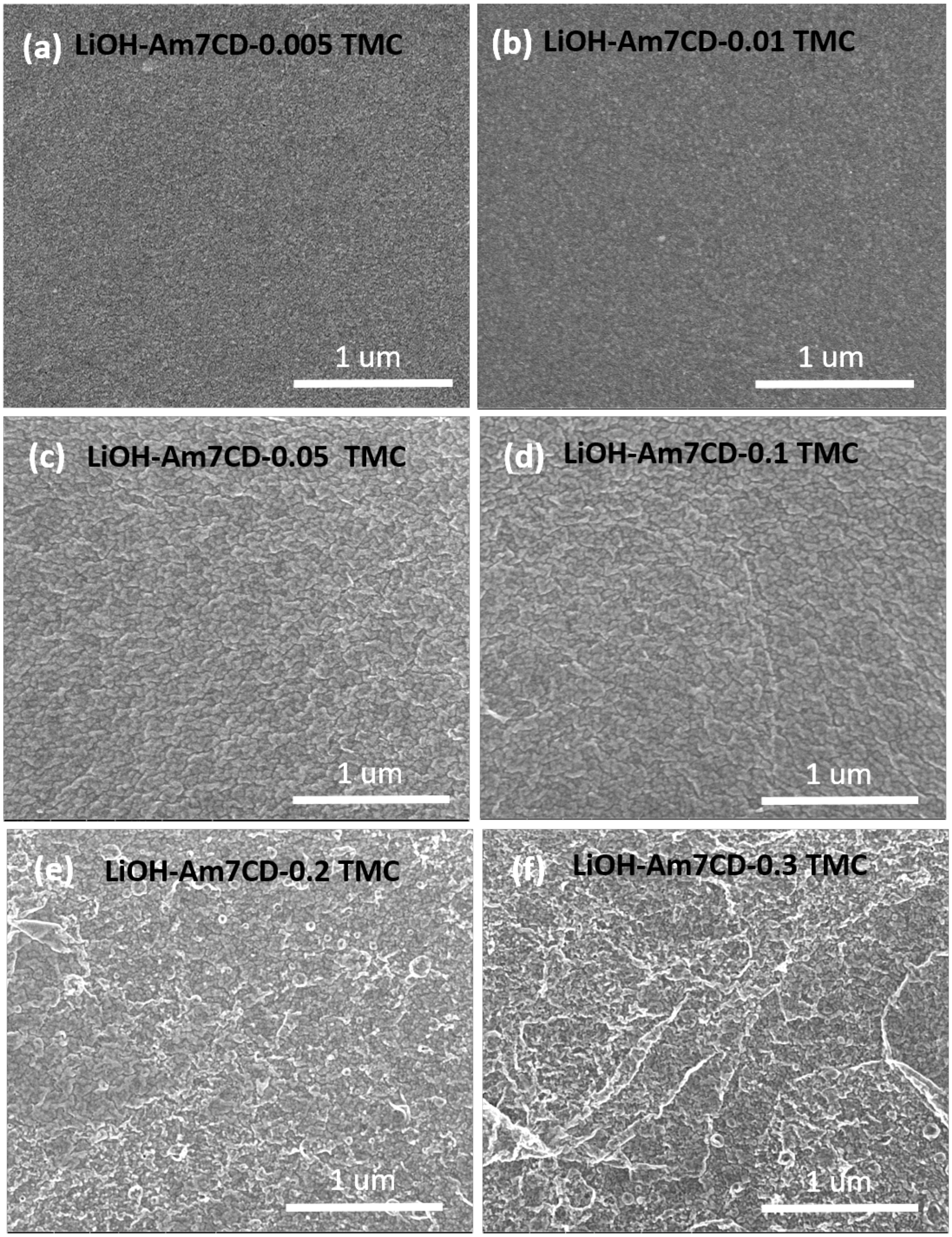

Supplementary Figure 9. SEM images depicting the surface morphology of LiOH-Am7CD-TMC membranes fabricated with various TMC concentrations: (a) 0.005% w/v, (b) 0.01% w/v, (c) 0.05% w/v, (d) 0.1% w/v, (e) 0.2% w/v, (f) 0.3% w/v. The detailed fabrication procedure of cyclodextrin-based TFC membranes is provided in the membrane preparation section.

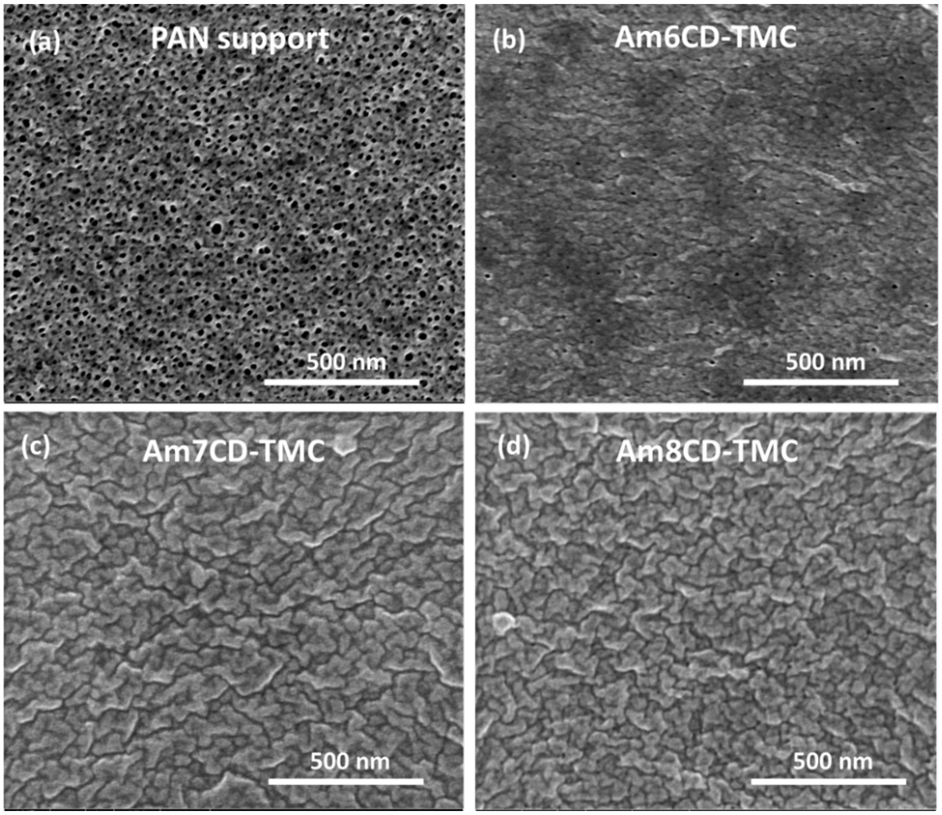

Supplementary Figure 10. SEM micrographs of the top-surface morphology of (a) PAN support; (b) LiOH-Am6CD-0.1 TMC membrane; (c) LiOH-Am7CD-0.1 TMC membrane and (d) LiOH-Am8CD-0.1 TMC membrane. All cyclodextrin-based TFC membranes were fabricated via interfacial polymerization on the PAN support layer.

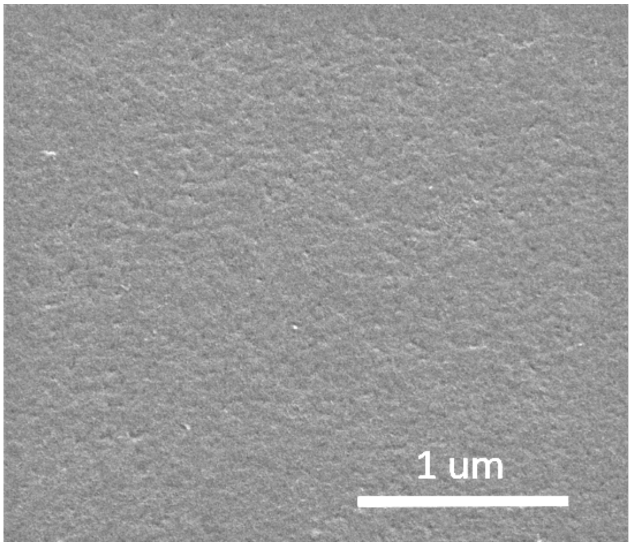

Supplementary Figure 11. SEM image of the top-surface morphology of HAc-Am7CD-0.3 TMC membrane fabricated on PAN support using a reaction time of 12 minutes.

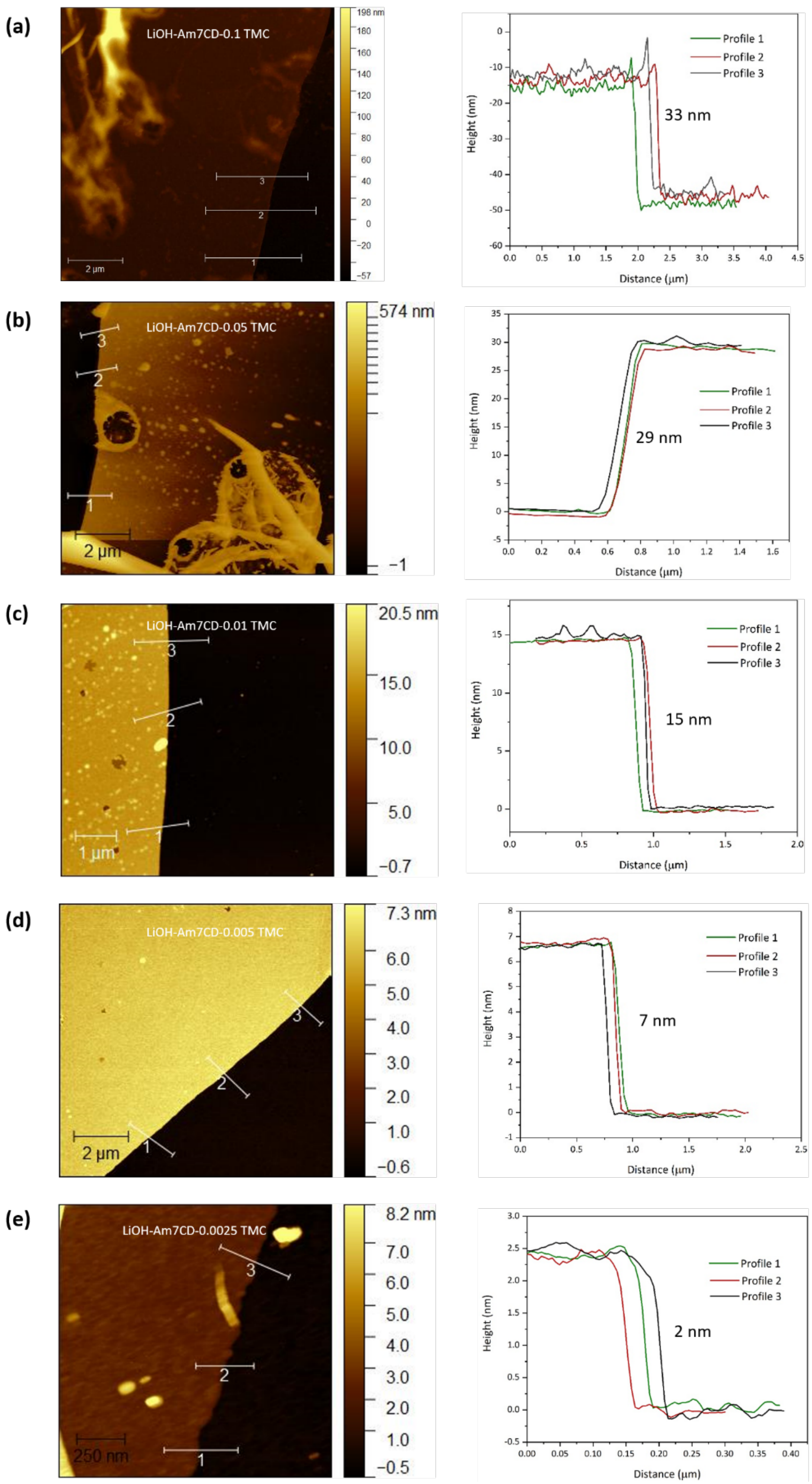

Supplementary Figure 12. 2D AFM images and corresponding extracted height profiles of LiOH-Am7CD-TMC membranes prepared using various TMC concentrations: (a) 0.1% w/v, (b) 0.05% w/v, (c) 0.01% w/v, (d) 0.005% w/v, (e) 0.0025% w/v.

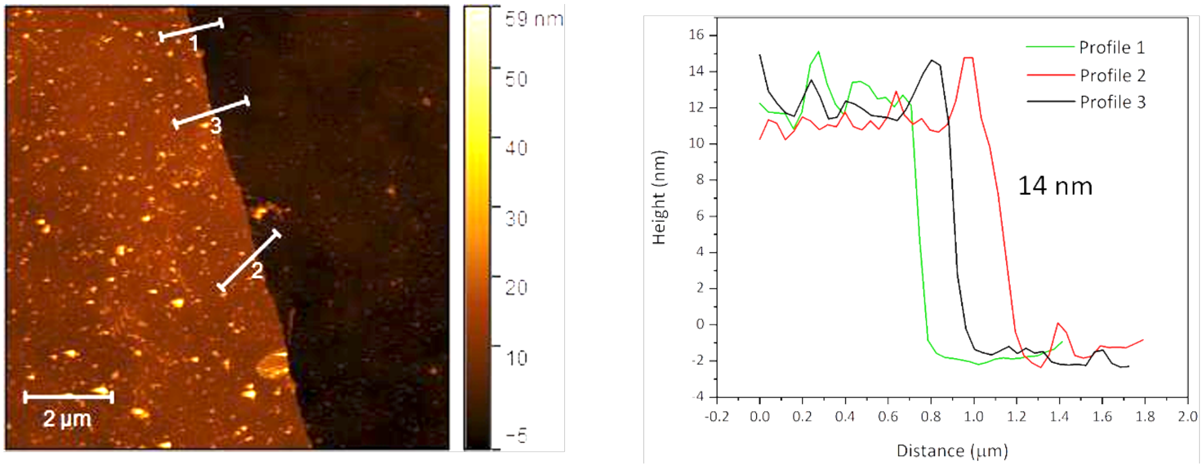

Supplementary Figure 13. 2D AFM image and extracted height profile of HAc-Am7CD-0.3 TMC membrane. For this analysis, the membrane was synthesized with a reaction time of 12 minutes.

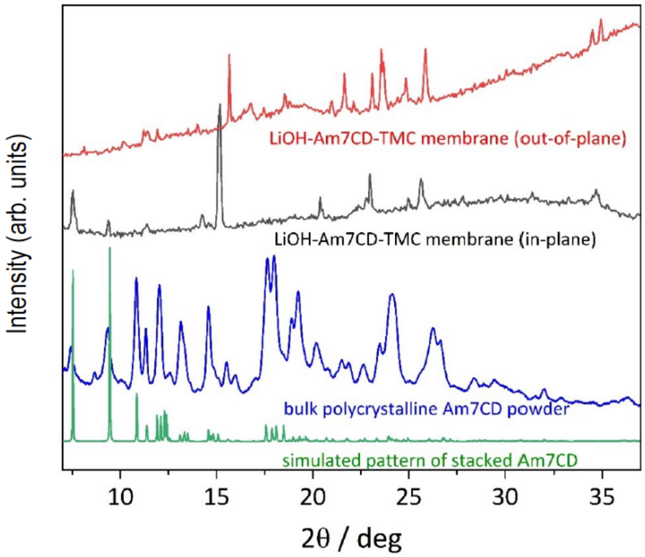

Supplementary Figure 14. Comparative spectral analysis including extracted GIWAXS one-dimensional spectra profiles (black and red curves) of LiOH-Am7CD-0.01 TMC membrane, PXRD results of polycrystalline Am7CD powder (blue curve) and the simulated pattern of stacked Am7CD (green curve).

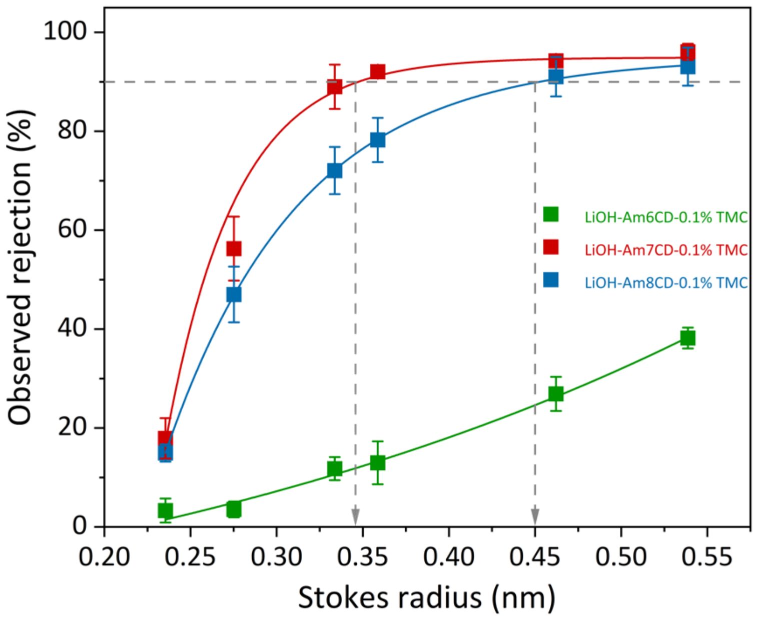

Supplementary Figure 15. Rejection profile of neutral solutes (including raffinose, sucrose, glucose, xylose, glycerol, and ethylene glycol) plotted as a function of the calculated solute Stokes radius for the three different types of cyclodextrin-based membranes. The grey horizontal dashed line indicates a rejection cut-off of 90%. Error bar standard deviation.

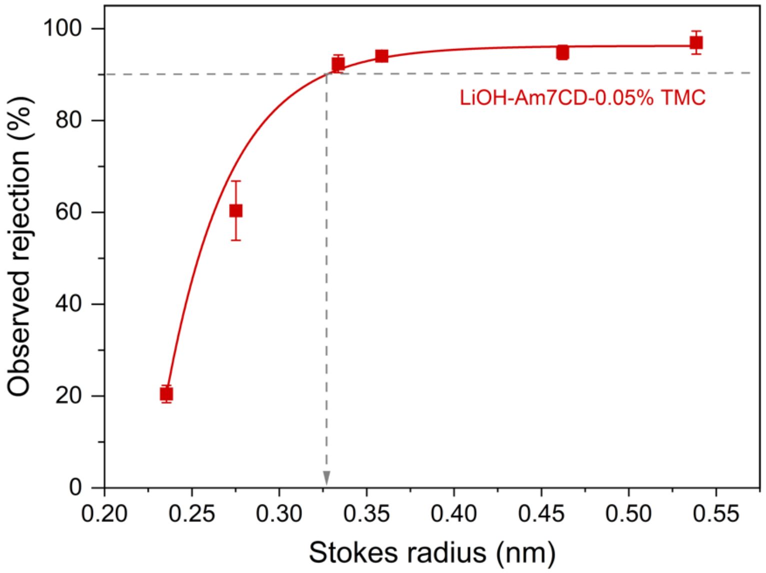

Supplementary Figure 16. Observed rejection of neutral solutes (including raffinose, sucrose, glucose, xylose, glycerol and ethylene glycol) plotted as a function of the calculated solute Stokes radius for LiOH-Am7CD-0.05 TMC membranes. The grey horizontal dash line indicates a rejection cut-off of 90%. Error bar standard deviation.

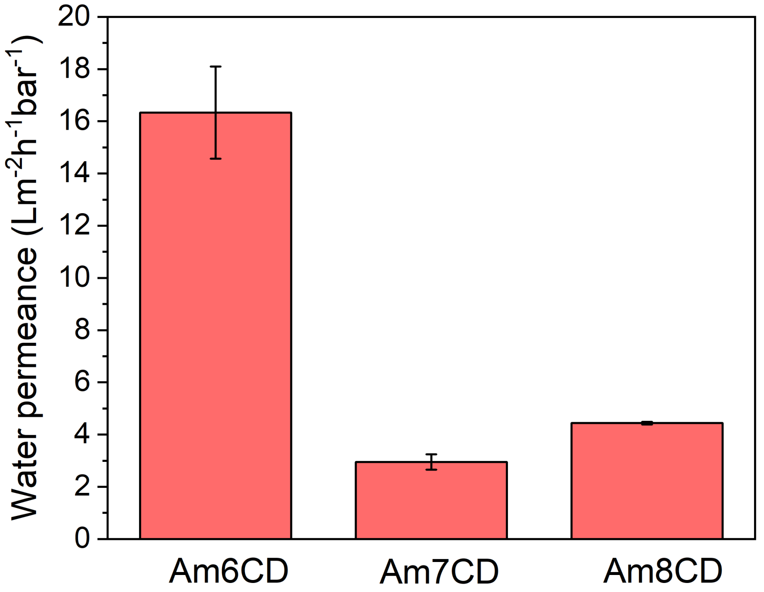

Supplementary Figure 17. Experimental water permeance for the three cyclodextrin-based composite membranes (namely, LiOH-Am6CD-0.1 TMC, LiOH-Am7CD-0.1 TMC, and LiOH-Am8CD-0.1 TMC) under 5 bar of applied pressure. Error bar standard deviation.

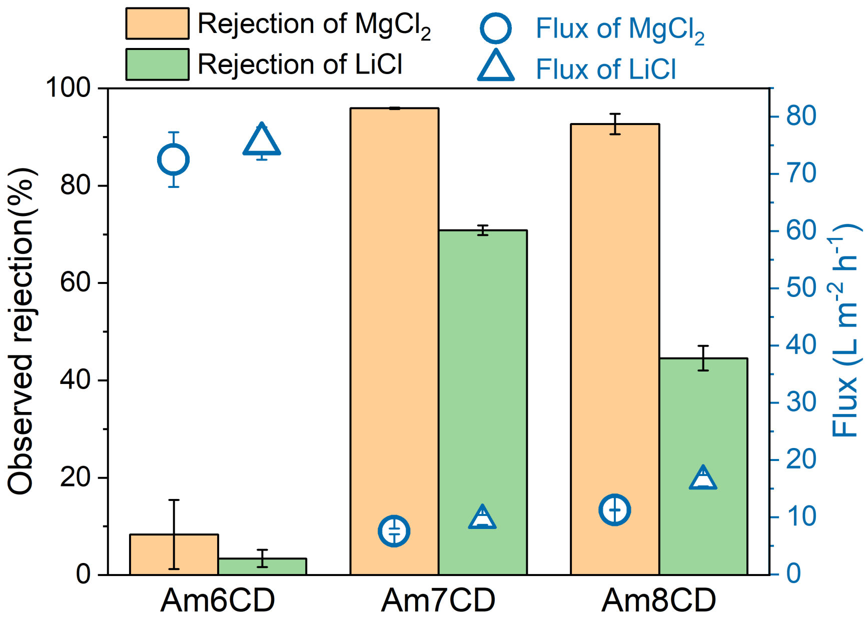

Supplementary Figure 18. Observed salt rejection (columns) and measured flux (symbols) for the three different types of cyclodextrin-based composite membranes (LiOH-Am6CD-0.1 TMC, LiOH-Am7CD-0.1 TMC, and LiOH-Am8CD-0.1 TMC). Filtration tests were conducted using feed solutions containing 2000 ppm $MgCl_2$ and 2000 ppm LiCl, respectively. Error bar standard deviation.

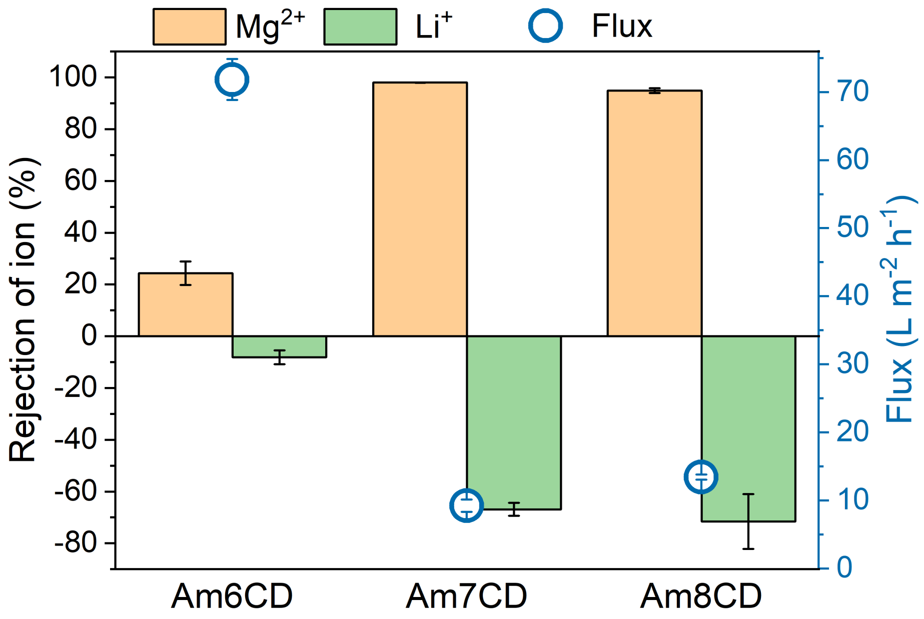

Supplementary Figure 19. Experimental ion rejection (column) and flux (empty circles) data for the three types of cyclodextrin-based composite membranes (LiOH-Am6CD-0.1 TMC, LiOH-Am7CD-0.1 TMC, and LiOH-Am8CD-0.1 TMC) using a feed containing 2000 ppm $MgCl_2$ and 100 ppm LiCl. Error bar standard deviation.

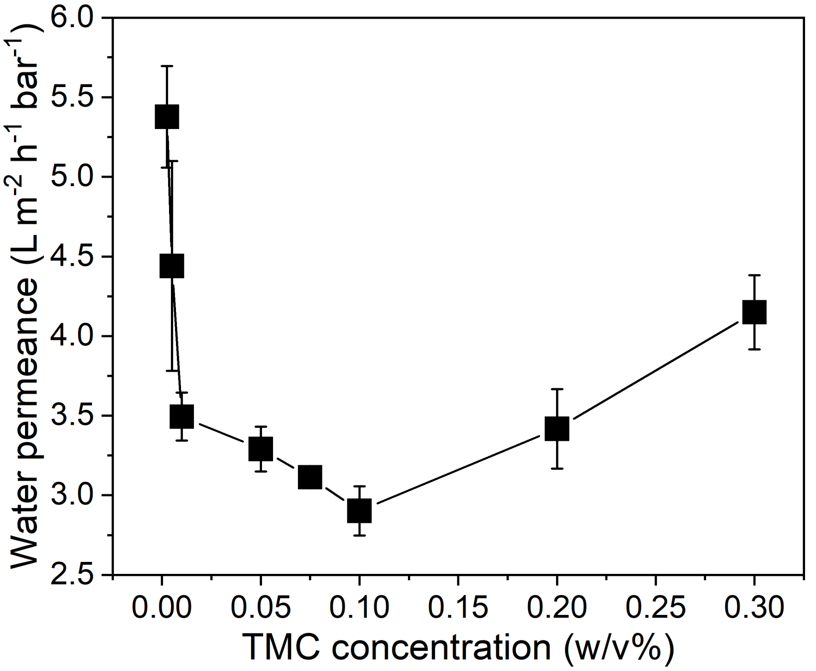

Supplementary Figure 20. Water permeance as a function of the TMC concentration used for fabricating LiOH-Am7CD-TMC membranes. The data were obtained under 6 bar of applied pressure and using distilled water as feed solution. Error bar standard deviation.

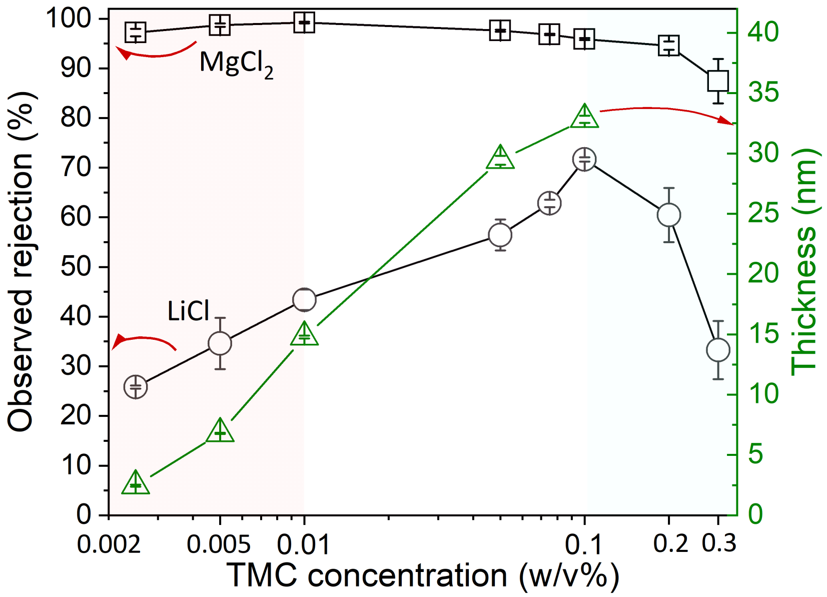

Supplementary Figure 21. Correlation between TMC concentration used during membrane fabrication, salt rejection (black curves with open symbols), and membrane thickness (green line with open triangles) for LiOH-Am7CD-TMC membranes. Filtration tests were performed using feed solutions containing either 2000 ppm $MgCl_2$ or 2000 ppm LiCl under 5 bar of applied pressure. Each membrane sample was analyzed three times by AFM to calculate the average thickness and the standard deviation (please refer to Supplementary Figure 12 for the AFM characterization). Error bar standard deviation.

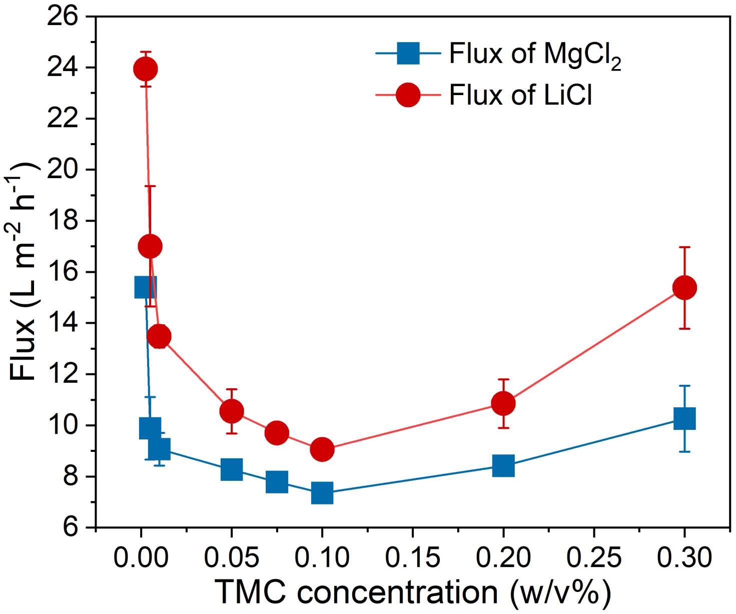

Supplementary Figure 22. Measured flux as a function of the TMC concentration used during LiOH-Am7CD-TMC membranes fabrication. Filtration tests were performed under 5 bar using feed solutions containing 2000 ppm $MgCl_2$ and 2000 ppm LiCl, respectively. Error bar standard deviation.

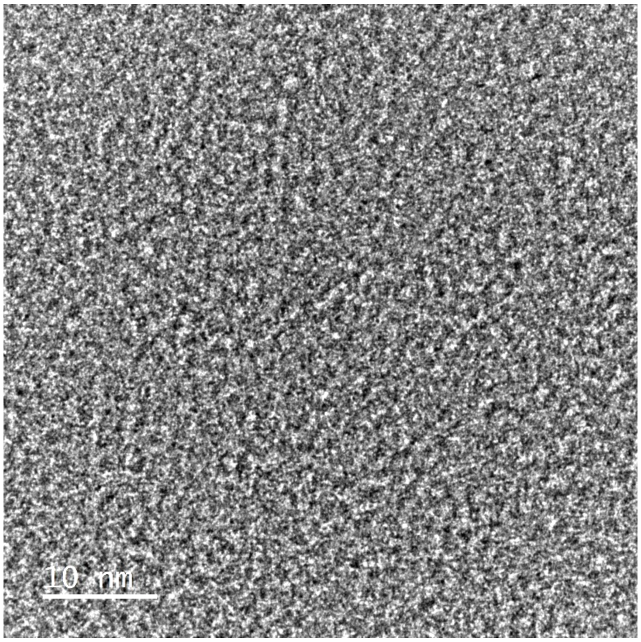

Supplementary Figure 23. TEM image of HAc-Am7CD-0.3 TMC membrane without lattice fringes. For this analysis, HAc-Am7CD-0.3 TMC membranes were prepared with an extended reaction time of 28 minutes to achieve an increased thickness and prevent possible damage from the electron stream.

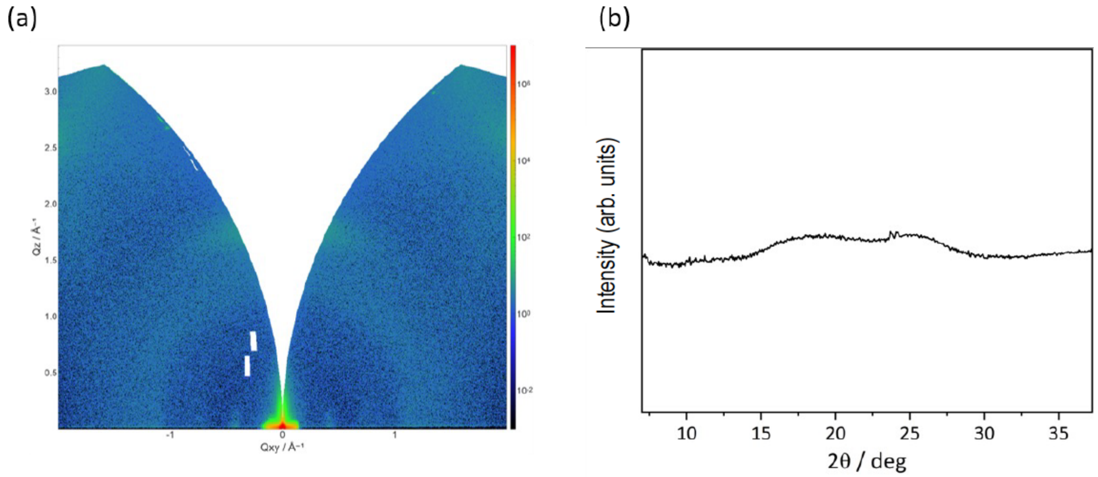

Supplementary Figure 24. GIWAXS analysis. (a) Two-dimensional pattern and (b) integrated one-dimensional spectra of HAc-Am7CD-0.3 TMC membranes fabricated with a reaction time of 12 minutes.

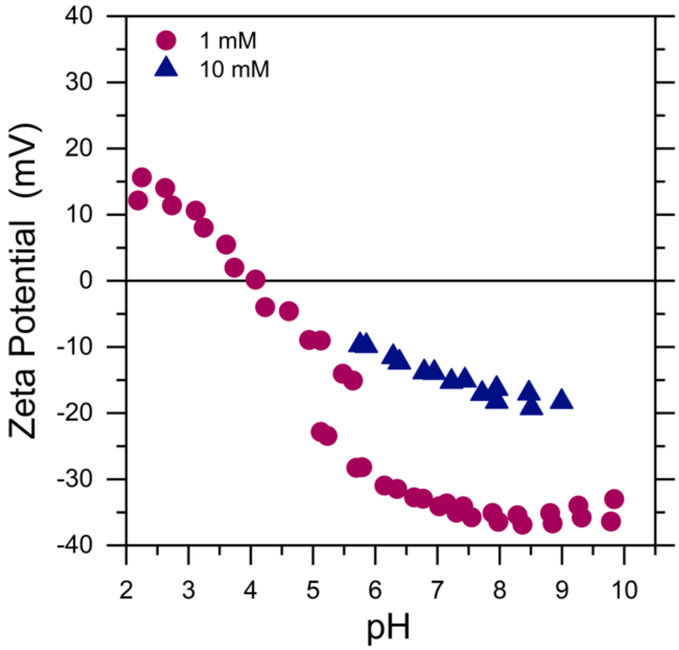

Supplementary Figure 25. Surface z-potential of LiOH-Am7CD-0.05 TMC membranes as a function of pH at low (1mM KCl, purple circles) and high (10 mM KCl, blue triangles) electrolyte concentrations.

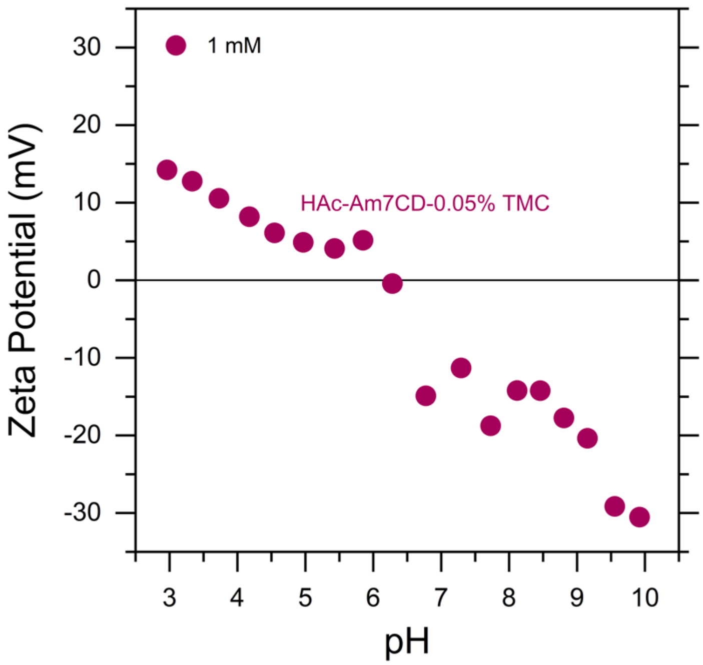

Supplementary Figure 26. Surface z-potential of HAc-Am7CD-0.3 TMC membrane plotted as a function of the pH using 1mM KCl electrolyte concentration.

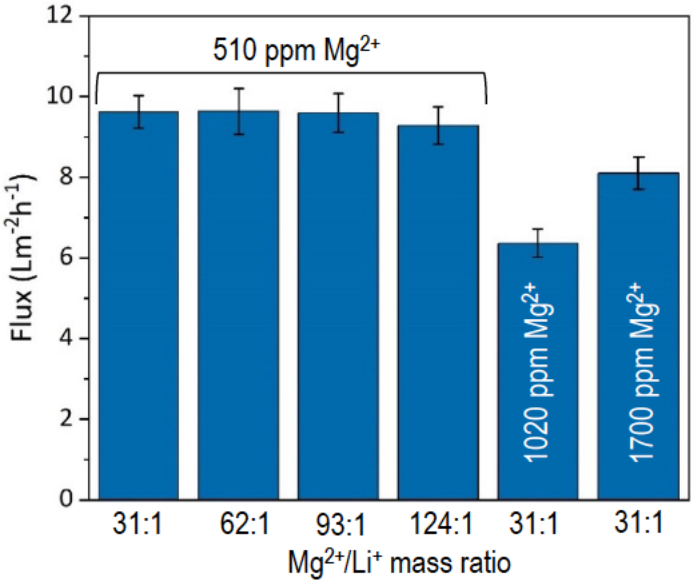

Supplementary Figure 27. Measured flux of LiOH-Am7CD-0.05% TMC membranes using a series of feed solutions with varying Mg^{2+}/Li^{+} mass ratios and Mg^{2+} concentrations. All filtration tests were conducted under 5 bar, except for the final one with a Mg^{2+} mass concentration of 1700 ppm, which was performed at 10 bar to overcome the osmotic pressure. Error bar standard deviation.

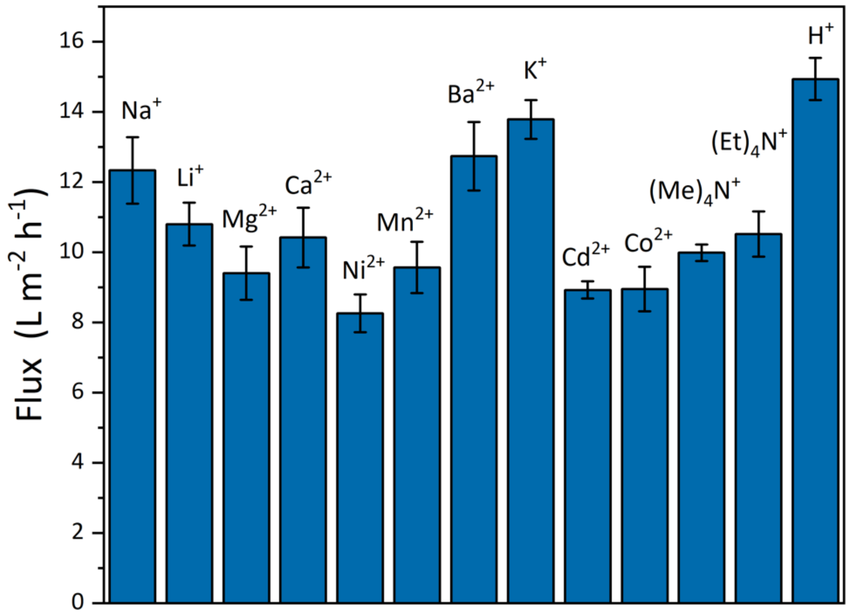

Supplementary Figure 28. Experimental flux of LiOH-Am7CD-0.05 TMC membranes during the filtration of diverse cations (with Cl^- as the counter-ion in the feeds). The experiments were performed using a series of single-salt solutions with a concentration of 2000 ppm. Error bar standard deviation.

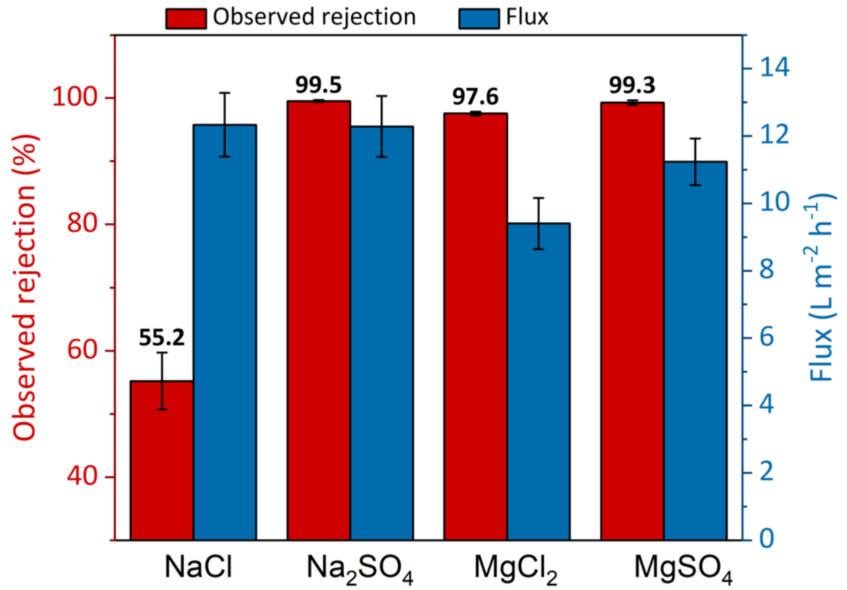

Supplementary Figure 29. Experimental observed salt rejection (red columns) and measured flux (blue columns) of LiOH-Am7CD-0.05 TMC membranes using single-salt feed solutions. Filtration tests were conducted under 5 bar of applied pressure using feed solutions containing 2000 ppm of NaCl, Na_2SO_4, $MgCl_2$ or $MgSO_4$, respectively. Error bar standard deviation.

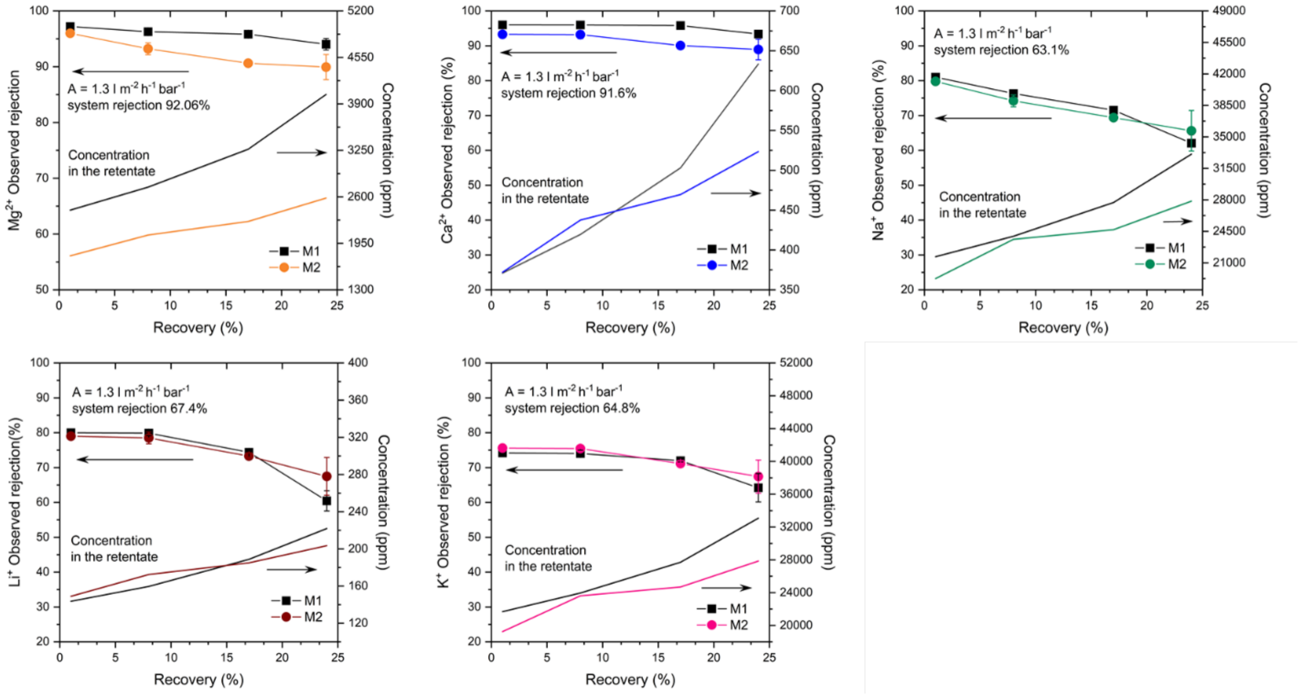

Supplementary Figure 30. Experimental ionic concentration in the retentate and corresponding observed rejection of Am7CD membranes plotted as a function of the recovery (0 to 25%) using a synthetic concentrated seawater brine solution. (Note: applied pressure was 70 bar).

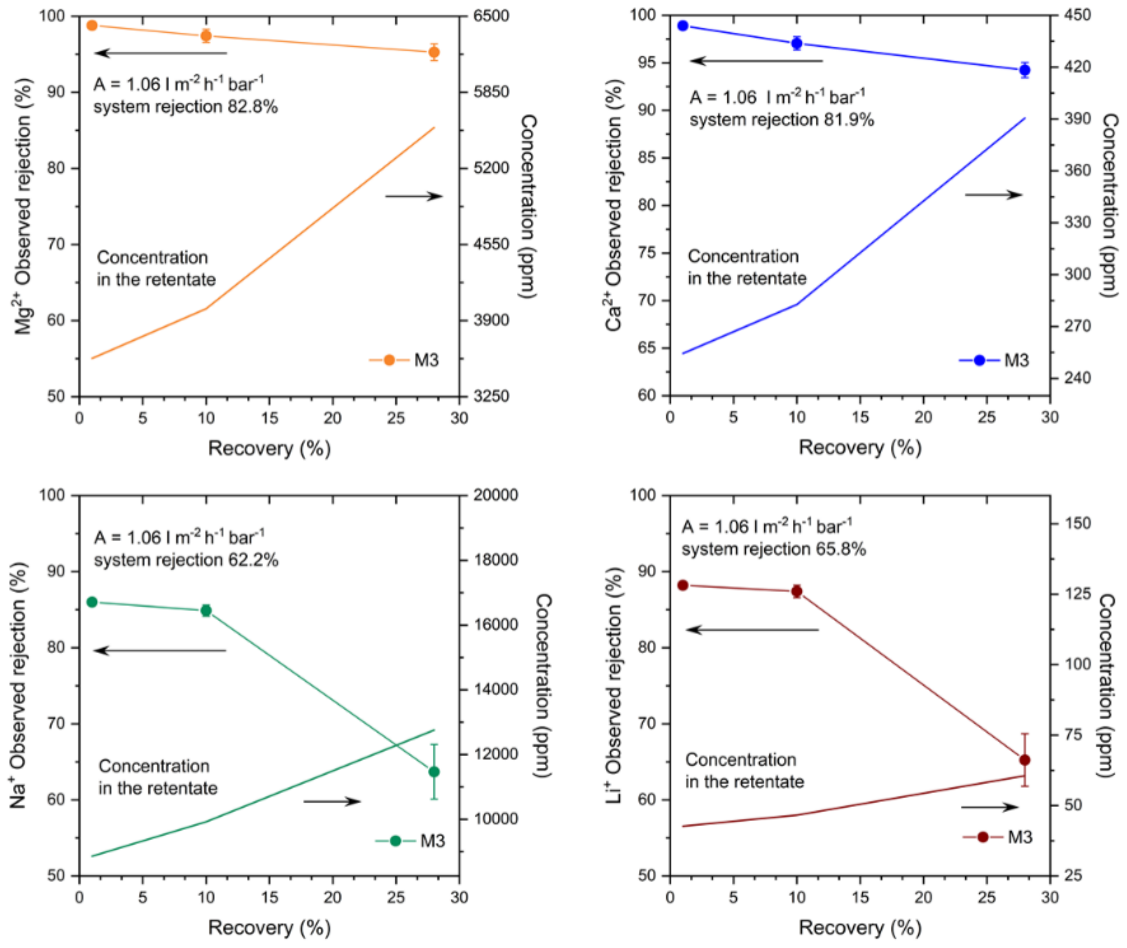

Supplementary Figure 31. Experimental ionic concentration in the retentate and corresponding observed rejection of Am7CD membranes plotted as a function of the recovery (0 to 30%) using a synthetic Lungmu Co salt lake brine solution. (Note: applied pressure was 60 bar). Error bar standard deviation.

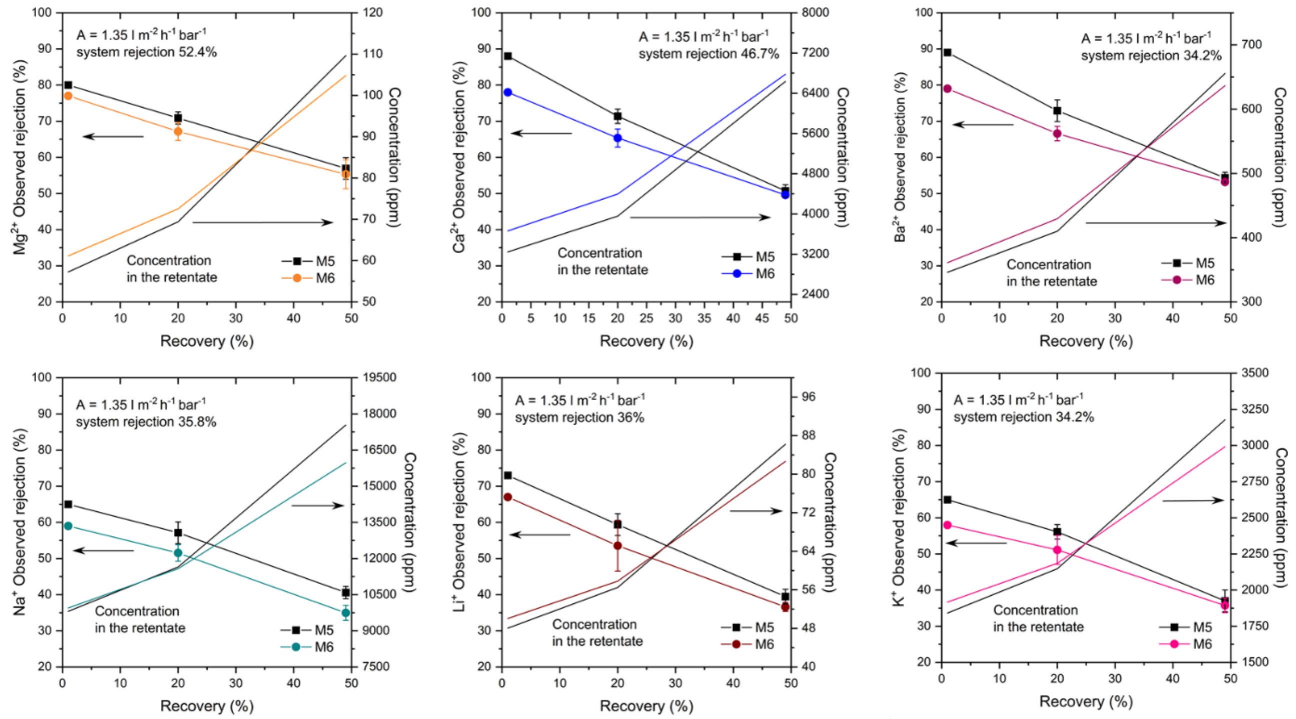

Supplementary Figure 32. Experimental ionic concentration in the retentate and corresponding observed rejection of Am7CD membranes plotted as a function of the recovery (0 to 50%) using a synthetic Imperial geothermal brine solution. (Note: applied pressure was 60 bar). Error bar standard deviation.

Supplementary Table 6. Comparison of separation factor and permeance between the LiOH-Am7CD-TMC membranes in this work and other state-of-the-art pressure-driven nanofiltration membranes.

Ref.	Membrane	Separation Factor (SF)	Permeance ($L \cdot m^{-2} \cdot h^{-1} \cdot bar^{-1}$)	Mg^{2+}/Li^{+} mass ratio in feed	Applied pressure (bar)	Membrane fabrication method
[8]	QBPD-PEI	5.2	13.6	50:1	6	IP
[9]	PEI-rCDs-TMC	10.8	4.86	30:1	4	IP
[10]	PEI-TMC-DHTAB	60.1	NA	20:1	4	IP
[11]	GLIP-EDA-TMC	27.38	10	20:1	6	Gas/liquid IP
[12]	PEI-TMC-TQAIL	23.2	13.3	15:1	5	IP
[13]	RIP-PEI-TMC-0.250	9.22	NA	20:1	15	Reversed IP
[14]	PSF-(CA-PEI)/PIP/TMC	50.7	18.6	27:1	10	IP
[15]	N-CPTC-TAEA	36.5	NA	31:1	10	IP
[16]	PSS/PAH-2.5bilayer	87.2	10	60:1	4	LBL
[17]	UIO-66-NH_2/PSF-2	78.6	4.472	31:1	10	IP
[18]	QEDTP NFM	15.6	18.8	120:1	6	IP
[19]	PEI@15C5-TMC	11.9	8	20:1	5	IP
[20]	DABIL-5 TFC NF	26.49	37.12 to pure water	NA	5	IP
[21]	Uio-66-NH_2-based TMC/PEI	33	30.6	20:1	4	IP
[22]	PES/PIP/TMC/PEI	33.4	12 to pure water	20:1	4	IP
[23]	SP_E-PEI600	12.37	NA	150:1	6	IP
[24]	(MWCNTs-COOK)-PEI/TMC	58	12.23	20:1	3	IP
[25]	DAIB	16.6	15.5	16:1	6	IP
[26]	Cu-MPD/TMC NF membrane	8	16.2	23:1	5	IP
[27]	PEI/TMC/CNC-COOH/PES	12.2	4.17	30:1	8	IP
[28]	[MimAP][Tf_2N]-PA/PAN	8.12	4.72	20:1	6	IP
[29]	PEI/TMC/(PES-GO)	16.1	11.15	20:1	3	IP
[30]	(PIP-PHF)/TMC/PES	13.1	6.3	21:1	6	IP
[31]	PEI/TMC/ PES	20	5.02 to pure water	20:1	8	IP

[32]	BPEI/TMC/EDTA	9.2	0.6	37:1	10	IP
[33]	PIP-MWCNTs/PEI/PES	16.5	9	21:1	4	IP
[34]	DAPP/TMC NF hollow fiber	2.6	2.6 to 2000 ppm $MgCl_2$	20:1	3	IP
[35]	MBCN-0.02	23.9	5.6 to 2000 ppm $MgCl_2$	73:1	4	IP
[36]	PIP-TMC/0.02% AB_2	35.7	12.7	21:1	10	IP
[37]	PIP/TMC-g-TETA	130	flux: 50 $L\cdot m^{-2}\cdot h^{-1}$	200:1	NA	IP
[38]	DTES/PEI/TMC	13	6.21	20:1	8	IP
[39]	$(PSS\text{-}PAH)_{2.5}$	382	8	60:1	4	LBL
[40]	M-SOH	11	23.8	100:1	6	IP
[41]	PA-g-AS-Fe	81.5	8.7	20:1	5	IP
[42]	SIP-0.15	15.38	1.3	20:1	15	IP
[43]	PEI/GQDs-NH_2/TMC	27.9	11.94	20:1	3	IP
[44]	PDA-PEI DK	60	3.37	30:1	NA	IP
[45]	MCPM-2.0	18	16 to pure water	1.6:1	2	IP
[46]	GEM-TMC	15	18.6	100:1	6	IP
[47]	PEI/Cyclen-TMC	8	14	20:1	5	IP
[48]	PAA-TMC	83	7.4	20:1	5	IP
This work	LiOH-Am7CD-0.05 TMC	190	1.92	31:1	5	IP
	LiOH-Am7CD-0.01 TMC	827	2.09			
	LiOH-Am7CD-0.005 TMC	256	2.53			

IP: Interfacial polymerization; LBL: Layer-by-layer.

Supplementary Table 7. Comparison of the Li^+/Mg^{2+} selectivity and Li^+ ion permeation rate (J_{Li+}) between our pressure-driven membranes and other state-of-the-art membranes operated under electrical potential or concentration gradients.

Ref.	Membrane	Li^+/Mg^{2+} selectivity	J_{Li+} ($mol \cdot m^{-2} \cdot h^{-1}$)	C_{Feed} ($mol \cdot L^{-1}$)	Driving force
49	PSS@HKUST-1	1815	6.75	0.5	Applied Voltage=0.4V
50	MXene@PSS	25	0.08	0.2	ΔC
51	Asy-MOFSNC Membrane *	1590	NA	0.1	Applied Voltage=1V
	Asy-MOFSNC Membrane	197.6	2.04×10^5	1	Applied Voltage=1V
52	PET Lumirror® membranes*	90	10.8	1	Applied Voltage=10V
	PET Lumirror® membranes	21.21	0.0143	0.5	Applied Voltage=10V
53	GO-PEI	21.9	0.306	0.5	ΔC
54	FRGO membranes	12	0.015	0.1	ΔC
55	$TpBDMe_2$ membranes*	217	0.0553	0.1	ΔC
	$TpBDMe_2$ membranes	35.8	0.0383	0.1	ΔC
56	$COF-EB_1BD_1/PAN$	353	NA	0.5	ΔC
	$COF-EB_1BD_1/PAN$	443	0.047	0.1	current density = 0.5 $mA \cdot cm^{-2}$
57	PCGO*	500	5.00×10^{-4}	1	ΔC
58	$UiO-66-SO_3H(25\%)$*	776	0.17	0.1	current density = 5 $mA \cdot cm^{-2}$

	UiO-66-SO_3H(25%)	1.88	0.1	0.1	current density = 5 mA·cm^{-2}
59	UiO-66-NH_2 LLM-4	65	0.072	0.1	current density = 5 mA·cm^{-2}
60	TFN-(Zr/Ti)-2	11.38	1.95	0.1	current density = 10 mA·cm^{-2}
61	CC3 membrane*	284	0.51	0.1	ED
	CC3 membrane	104	0.077	0.1	ED
62	$Ti_3C_2T_x$ Mxene*	8.75	1.4	0.2	ΔC
63	UiO-66-COOH-SNC*	260	NA	0.1	Applied Voltage=1V
64	PIM-EA-TB*	7.53	6.63	1	ΔC
	PIM-BzMA-TB*	32.6	1.27	1	ΔC
	DMBP-TB*	14.6	0.06	1	ΔC
	AO-PIM-1*	16.1	0.61	1	ΔC
This work	LiOH-Am7CD-0.05 TMC	190	0.035	0.02	ΔP=5bar
	LiOH-Am7CD-0.01 TMC	827	0.0295		
	LiOH-Am7CD-0.005 TMC	256	0.0355		

* Feed with single-salt solution compositions.

All others have binary- or multiple-salt solution feed.

Feed solutions in this work: 2000 ppm $MgCl_2$ and 100 ppm LiCl, corresponding to a total concentration of 0.02 mol·L^{-1}.

In our study, J_{Li+} is defined as the molar quantity of Li^+ ions that traverse a 1 m^2 membrane over one hour under the operational pressure. Calculation is represented by Equation (6):

$$J_{Li+} = \frac{C_{Li,P}}{1000\times M(Li)} \times A \times \Delta P \quad (6)$$

Where:

- $C_{Li,P}$ (ppm) denotes the concentration of Li^+ ions in the collected permeate liquid, determined using ICP-OES analysis.
- M(Li) represents the atomic mass of lithium, a constant value of 6.941g/mol.
- A and ΔP correspond to permeance and applied pressure, respectively, in the pressure-driven nanofiltration tests using a dead-end cell.

For instance, when calculating J_{Li+} for the LiOH-Am7CD-0.01 TMC membrane, with a Li^+ ion concentration of 19.57 ppm in the permeate side, and A and ΔP values of 2.09 L m^{-2} h^{-1} bar^{-1} and 5 bar, respectively, the calculation yields: $J_{Li+} = \frac{19.57}{1000 \times 6.941} \times 2.09 \times 5 = 0.0295$ mol m^{-2} h^{-1}.

References

1 Guillo, F. *et al.* Synthesis of symmetrical cyclodextrin derivatives bearing multiple charges. *Bulletin de la Société chimique de France* **8**, 857-866 (1995).

2 Okamatsu, A. *et al.* Design and evaluation of folate-appended α-, β-, and γ-cyclodextrins having a caproic acid as a tumor selective antitumor drug carrier in vitro and in vivo. *Biomacromolecules* **14**, 4420-4428 (2013).

3 Ashton, P. R., Königer, R., Stoddart, J. F., Alker, D. & Harding, V. D. Amino acid derivatives of β-cyclodextrin. *The journal of organic chemistry* **61**, 903-908 (1996).

4 Huang, T., Puspasari, T., Nunes, S. P. & Peinemann, K. V. Ultrathin 2D‐layered cyclodextrin membranes for high‐performance organic solvent nanofiltration. *Advanced Functional Materials* **30**, 1906797 (2020).

5 Bowen, W. & Mohammad, A. W. Characterization and prediction of nanofiltration membrane performance—a general assessment. *Chemical Engineering Research and Design* **76**, 885-893 (1998).

6 Giagnorio, M. *et al.* Achieving low concentrations of chromium in drinking water by nanofiltration: membrane performance and selection. *Environmental Science and Pollution Research* **25**, 25294-25305 (2018).

7 Giagnorio, M., Ricceri, F. & Tiraferri, A. Desalination of brackish groundwater and reuse of wastewater by forward osmosis coupled with nanofiltration for draw solution recovery. *Water research* **153**, 134-143 (2019).

8 Feng, Y., Peng, H. & Zhao, Q. Fabrication of high performance Mg2+/Li+ nanofiltration membranes by surface grafting of quaternized bipyridine. *Separation and Purification Technology* **280**, 119848 (2022).

9 Zhao, Y. *et al.* Extra-thin composite nanofiltration membranes tuned by γ-cyclodextrins containing amphipathic cavities for efficient separation of magnesium/lithium ions. *Separation and Purification Technology* **286**, 120419 (2022).

10 Gu, T. *et al.* Quaternary ammonium engineered polyamide membrane with high positive charge density for efficient Li+/Mg2+ separation. *Journal of Membrane Science* **659**, 120802 (2022).

11 Wu, M.-B. *et al.* Positively-charged nanofiltration membranes constructed via gas/liquid interfacial polymerization for Mg2+/Li+ separation. *Journal of Membrane Science* **644**, 119942 (2022).

12 Soyekwo, F., Wen, H., Liao, D. & Liu, C. Fouling-resistant ionic graft-polyamide nanofiltration membrane with improved permeance for lithium separation from MgCl2/LiCl mixtures. *Journal of Membrane Science* **659**, 120773 (2022).

13 Li, Y. *et al.* Fabrication of positively charged nanofiltration membrane with uniform charge distribution by reversed interfacial polymerization for Mg2+/Li+ separation. *Journal of Membrane Science* **659**, 120809 (2022).

14 Chen, K. *et al.* Dual-electric layer nanofiltration membranes based on polyphenol/PEI interlayer for highly efficient Mg2+/Li+ separation. *Journal of Membrane Science* **660**, 120860 (2022).

15 Yuan, B. *et al.* Aliphatic polyamide nanofilm with ordered nanostripe, synergistic pore size and charge density for the enhancement of cation sieving. *Journal of Membrane Science* **660**, 120839 (2022).

16 He, R. *et al.* Unprecedented Mg2+/Li+ separation using layer-by-layer based nanofiltration hollow fiber membranes. *Desalination* **525**, 115492 (2022).

17 Yuan, B. *et al.* Polyamide nanofiltration membrane fine-tuned via mixed matrix ultrafiltration support to maximize the sieving selectivity of Li+/Mg2+ and Cl–/SO42–. *Desalination* **538**, 115929 (2022).

18 Xu, Y. *et al.* High performance Mg2+/Li+ separation membranes modified by a bis-quaternary ammonium salt. *Desalination* **526**, 115519 (2022).

19 Li, H. *et al.* Nanofiltration membrane with crown ether as exclusive Li+ transport channels achieving efficient extraction of lithium from salt lake brine. *Chemical Engineering Journal* **438**, 135658 (2022).

20 Soyekwo, F., Wen, H., Liao, D. & Liu, C. Nanofiltration membranes modified with a clustered multiquaternary ammonium-based ionic liquid for improved magnesium/lithium separation. *ACS Applied Materials & Interfaces* **14**, 32420-32432 (2022).

21 Aghili, F., Ghoreyshi, A. A., Van der Bruggen, B. & Rahimpour, A. A highly permeable UiO-66-NH2/polyethyleneimine thin-film nanocomposite membrane for recovery of valuable metal ions from brackish water. *Process Safety and Environmental Protection* **151**, 244-256 (2021).

22 Yang, Z. *et al.* Dual-skin layer nanofiltration membranes for highly selective Li+/Mg2+ separation. *Journal of Membrane Science* **620**, 118862 (2021).

23 Lu, D. *et al.* Constructing a selective blocked-nanolayer on nanofiltration membrane via surface-charge inversion for promoting Li+ permselectivity over Mg2+. *Journal of Membrane Science* **635**, 119504 (2021).

24 Xu, P., Hong, J., Xu, Z., Xia, H. & Ni, Q.-Q. MWCNTs-COOK-assisted high positively charged composite membrane: Accelerating Li+ enrichment and Mg2+ removal. *Composites Part B: Engineering* **212**, 108686 (2021).

25 Peng, H. & Zhao, Q. A nano‐heterogeneous membrane for efficient separation of lithium from high magnesium/lithium ratio brine. *Advanced Functional Materials* **31**, 2009430 (2021).

26 Wang, L. *et al.* Novel positively charged metal-coordinated nanofiltration membrane for lithium recovery. *ACS Applied Materials & Interfaces* **13**, 16906-16915 (2021).

27 Guo, C. *et al.* Ultra-thin double Janus nanofiltration membrane for separation of Li+ and Mg2+:“Drag” effect from carboxyl-containing negative interlayer. *Separation and Purification Technology* **230**, 115567 (2020).

28 Wu, H. *et al.* A novel nanofiltration membrane with [MimAP][Tf2N] ionic liquid for utilization of lithium from brines with high Mg2+/Li+ ratio. *Journal of Membrane Science* **603**, 117997 (2020).

29 Xu, P. *et al.* “Bridge” graphene oxide modified positive charged nanofiltration thin membrane with high efficiency for Mg2+/Li+ separation. *Desalination* **488**, 114522 (2020).

30 Shen, Q., Xu, S. J., Xu, Z. L., Zhang, H. Z. & Dong, Z. Q. Novel thin‐film nanocomposite membrane with water‐soluble polyhydroxylated fullerene for the separation of Mg2+/Li+ aqueous solution. *Journal of Applied Polymer Science* **136**, 48029 (2019).
31 Xu, P. *et al.* Positive charged PEI-TMC composite nanofiltration membrane for separation of Li+ and Mg2+ from brine with high Mg2+/Li+ ratio. *Desalination* **449**, 57-68 (2019).
32 Li, W. *et al.* A positively charged composite nanofiltration membrane modified by EDTA for LiCl/MgCl2 separation. *Separation and Purification Technology* **186**, 233-242 (2017).
33 Zhang, H.-Z., Xu, Z.-L., Ding, H. & Tang, Y.-J. Positively charged capillary nanofiltration membrane with high rejection for Mg2+ and Ca2+ and good separation for Mg2+ and Li+. *Desalination* **420**, 158-166 (2017).
34 Li, X. *et al.* Preparation and characterization of positively charged polyamide composite nanofiltration hollow fiber membrane for lithium and magnesium separation. *Desalination* **369**, 26-36 (2015).
35 Bi, Q., Zhang, C., Liu, J., Liu, X. & Xu, S. Positively charged zwitterion-carbon nitride functionalized nanofiltration membranes with excellent separation performance of Mg2+/Li+ and good antifouling properties. *Separation and purification technology* **257**, 117959 (2021).
36 Hu, P. *et al.* Modification of polyamide nanofiltration membrane with ultra-high multivalent cations rejections and mono-/divalent cation selectivity. *Desalination* **527**, 115553 (2022).
37 Li, Q. *et al.* High performance Li+/Mg2+ separation membrane by grafted short chain amino-rich monomers. *Journal of Membrane Science* **677**, 121634 (2023).
38 Wu, H. *et al.* Positively-charged PEI/TMC nanofiltration membrane prepared by adding a diamino-silane coupling agent for Li+/Mg2+ separation. *Journal of Membrane Science* **672**, 121468 (2023).
39 He, R. *et al.* Polyelectrolyte-based nanofiltration membranes with exceptional performance in Mg2+/Li+ separation in a wide range of solution conditions. *Journal of Membrane Science* **663**, 121027 (2022).
40 Peng, H., Hu, Y., Li, S., Rao, J. & Zhao, Q. Sulfonium-polyamide membranes for high flux Mg2+/Li+ separation. *Journal of Membrane Science* **674**, 121515 (2023).
41 Liu, Y. *et al.* A nanofiltration membrane with positively and negatively charged groups by grafted p-aminosalicylic acid-Fe (III) chelation for Li+/Mg2+ efficient separation. *Separation and Purification Technology* **308**, 122968 (2023).
42 Li, Y. *et al.* Polyamide nanofiltration membranes with rigid–flexible microstructures for high-efficiency Mg2+/Li+ separation. *Separation and Purification Technology* **306**, 122552 (2023).
43 Xu, P., Hong, J., Xu, Z., Xia, H. & Ni, Q.-Q. Novel aminated graphene quantum dots (GQDs-NH2)-engineered nanofiltration membrane with high Mg2+/Li+ separation efficiency. *Separation and Purification Technology* **258**, 118042 (2021).
44 Ashraf, M. A. *et al.* Enhancement in Li+/Mg2+ separation from salt lake brine with PDA–PEI composite nanofiltration membrane. *Journal of Applied Polymer Science* **137**, 49549 (2020).
45 Zhao, J. *et al.* Mix-charged polyamide membranes via molecular hybridization for selective ionic nanofiltration. *Journal of Membrane Science* **644**, 120051 (2022).

46 Peng, H., Su, Y., Liu, X., Li, J. & Zhao, Q. Designing Gemini ‐ Electrolytes for Scalable Mg2+/Li+ Separation Membranes and Modules. *Advanced Functional Materials*, 2305815 (2023).

47 Li, T. *et al.* Nanofiltration membrane comprising structural regulator Cyclen for efficient Li+/Mg2+ separation. *Desalination* **556**, 116575 (2023).

48 Xu, P. *et al.* Fabrication of highly positively charged nanofiltration membranes by novel interfacial polymerization: Accelerating Mg2+ removal and Li+ enrichment. *Journal of Membrane Science* **668**, 121251 (2023).

49 Guo, Y., Ying, Y., Mao, Y., Peng, X. & Chen, B. Polystyrene sulfonate threaded through a metal ‐ organic framework membrane for fast and selective lithium ‐ ion separation. *Angewandte Chemie* **128**, 15344-15348 (2016).

50 Lu, Z., Wu, Y., Ding, L., Wei, Y. & Wang, H. A lamellar MXene (Ti3C2Tx)/PSS composite membrane for fast and selective lithium ‐ ion separation. *Angewandte Chemie* **133**, 22439-22443 (2021).

51 Lu, J. *et al.* Efficient metal ion sieving in rectifying subnanochannels enabled by metal–organic frameworks. *Nature materials* **19**, 767-774 (2020).

52 Wang, P. *et al.* Ultrafast ion sieving using nanoporous polymeric membranes. *Nature communications* **9**, 569 (2018).

53 Huang, Q., Liu, S., Guo, Y., Liu, G. & Jin, W. Separation of mono-/di-valent ions via charged interlayer channels of graphene oxide membranes. *Journal of Membrane Science* **645**, 120212 (2022).

54 Xi, Y.-H. *et al.* Graphene-based membranes with uniform 2D nanochannels for precise sieving of mono-/multi-valent metal ions. *Journal of Membrane Science* **550**, 208-218 (2018).

55 Sheng, F. *et al.* Efficient Ion Sieving in Covalent Organic Framework Membranes with Sub ‐ 2 ‐ Nanometer Channels. *Advanced Materials* **33**, 2104404 (2021).

56 Hou, L. *et al.* Understanding the ion transport behavior across nanofluidic membranes in response to the charge variations. *Advanced Functional Materials* **31**, 2009970 (2021).

57 Abraham, J. *et al.* Tunable sieving of ions using graphene oxide membranes. *Nature nanotechnology* **12**, 546-550 (2017).

58 Xu, T. *et al.* Engineering leaf-like UiO-66-SO 3 H membranes for selective transport of cations. *Nano-micro letters* **12**, 1-11 (2020).

59 Xu, T. *et al.* Highly Cation Permselective Metal ‐ Organic Framework Membranes with Leaf ‐ Like Morphology. *ChemSusChem* **12**, 2593-2597 (2019).

60 Xu, T. *et al.* Ti-exchanged UiO-66-NH2–containing polyamide membranes with remarkable cation permselectivity. *Journal of Membrane Science* **615**, 118608 (2020).

61 Xu, T. *et al.* Highly ion-permselective porous organic cage membranes with hierarchical channels. *Journal of the American Chemical Society* **144**, 10220-10229 (2022).

62 Ren, C. E. *et al.* Charge-and size-selective ion sieving through Ti3C2T x MXene membranes. *The journal of physical chemistry letters* **6**, 4026-4031 (2015).

63 Lu, J. *et al.* Ultraselective monovalent metal ion conduction in a three-dimensional sub-1 nm nanofluidic device constructed by metal–organic frameworks. *ACS nano* **15**, 1240-1249 (2020).

64 Tan, R. *et al.* Hydrophilic microporous membranes for selective ion separation and flow-battery energy storage. *Nature Materials* **19**, 195-202 (2020).
